# Role of Neural (N)-Cadherin in Breast Cancer Cell Stemness and Dormancy in the Bone Microenvironment

**DOI:** 10.3390/cancers14051317

**Published:** 2022-03-04

**Authors:** Antonio Maurizi, Michela Ciocca, Cristiano Giuliani, Ilaria Di Carlo, Anna Teti

**Affiliations:** Department of Biotechnological and Applied Clinical Sciences, University of L’Aquila, 67100 L’Aquila, Italy; michela.ciocca@graduate.univaq.it (M.C.); cristiano.giuliani@graduate.univaq.it (C.G.); ilaria.dicarlo1@guest.univaq.it (I.D.C.); annamaria.teti@univaq.it (A.T.)

**Keywords:** BrCa dormancy, cancer stem cells, HSC-mimicry, osteoblasts, endosteal niche, bone microenvironment

## Abstract

**Simple Summary:**

Breast cancer (BrCa) patients experience tumour recurrence 7–25 years after mastectomy. Dormant BrCa cells often home to the bone and, upon reactivation, lead to secondary lesions. We previously demonstrated that, in the bone marrow, dormant BrCa cells are located near the endosteal niche enriched in spindle-shaped N-cadherin-high Osteoblasts (SNOs), show a stem signature like the LT-HSCs and express high levels of Notch2. Here, we observed that Notch2^High^ human BrCa MDA cells are also N-Cadherin^High^. When injected in vivo, N-Cadherin^High^ MDA cells show lower aggressiveness, and higher ability to colonize the endosteal niche and to adhere to SNOs compared to N-Cadherin^Low^ MDA cells. These cells have features typical of cancer stem cells, displaying a gene signature similar to the HSCs. In contrast, N-cadherin has a negative role in mouse 4T1 cell stemness and HSC mimicry. In brief, our results identified a role of N-Cadherin in BrCa dormancy and stemness, also highlighting the differences between human and mouse BrCa cell lines.

**Abstract:**

Breast cancer cells that interact with spindle-shaped N-Cadherin^+^ Osteoblasts (SNOs) are recognised to become dormant through a Notch2-dependent mechanism. We found that Notch2^High^ human BrCa MDA-MB231 (MDA) cells also expressed high level of N-Cadherin. This prompted us to hypothesize that N-Cadherin could have a role in MDA-SNO interaction. Of note, the expression of N-Cadherin in MDA cells reduced tumour incidence and bone osteolysis in BrCa mouse model. Moreover, similarly to Notch2^High^ MDA cells, the N-Cadherin^High^ MDA cells revealed a high expression of the canonical Haematopoietic Stem cell (HSC) markers, suggesting an HSC mimicry, associated with higher ability to form mammospheres. Interestingly, N-Cadherin^High^ MDA cells showed greater capacity to adhere to SNOs, while the inhibition of SNO-mediating MDA cell proliferation was unremarkable. To investigate whether these features were shared by mouse BrCa, we used the 4T1 cell line in which N-Cadherin expression was abolished and then rescued. At variance with MDA cells, 4T1 cells expressing N-Cadherin revealed that the latter was associated with a lower expression of the HSC marker, Cxcr4, along with a lower capacity to form mammospheres. Furthermore, the rescue of N-Cadherin expression increased cell-cell adhesion and reduced proliferation of 4T1 cells when they were co-plated with SNOs. In conclusion, we demonstrated that: (i) N-Cadherin^High^ and Notch2^High^ MDA cells showed similar HSC mimicry and dormancy features; (ii) N-Cadherin mediated BrCa-SNO adhesion; (iii) N-Cadherin had a positive Notch2-dependent role on SNO-induced dormancy and HSC mimicry in MDA cells, and a negative role in 4T1 cell stemness and HSC mimicry.

## 1. Introduction

Breast cancer (BrCa) represents the second most common malignancy in women, with thousands of new cases diagnosed every year [1] and a five-year survival rate around 90% [1]. It is estimated that 20–45% of “disease-free” patients experience tumour recurrence 7–25 years after mastectomy [1,2]. This has been correlated with the ability of a subpopulation of BrCa cells to survive in the host in a dormant state [3]. Dormant cells are resistant to conventional therapies and are difficult to detect, making their eradication a real challenge in clinical practice [4,5]. The dormancy of BrCa cells is an unharmful event until these cells reactivate and initiate secondary tumours. Therefore, dormancy and tumour initiation ability, also known as tumour cell stemness, must co-exist in the same cancer cell. Indeed, recent studies have demonstrated that dormant breast cancer cells have the added ability to initiate new tumours, displaying cellular and molecular features typical of stem cells [6,7].

The bone marrow microenvironment has been identified as the main site in which BrCa dormant cells are lodged and kept in a quiescence status by specific signals and cell-cell interactions [8]. Bone marrow is known to contribute to the maintenance of long-term haematopoetic stem cell (LT-HSC) stemness and quiescence through specific niches. One of these niches is represented by a specialized subtype of osteoblasts, lining the endosteal surface and expressing high levels of Neural (N)-Cadherin, named spindle-shaped N-Cadherin^+^/CD45^−^ osteoblasts (SNOs) [9,10].

Recent work has established that the endosteal niche is also implicated in BrCa cell dormancy [11], and we have demonstrated that dormant BrCa cells, expressing a high level of N-Cadherin and Notch2, are in the proximity of the endosteal niche enriched in SNOs [12]. Our work suggested that SNOs keep the tumour cells dormant by a mechanism such as that of LT-HSC quiescence [12]. Moreover, this BrCa subpopulation shows a stem signature similar to LT-HSCs [12], thus suggesting an HSC mimicry.

N-Cadherin is a member of the cadherin family, mediating homophilic cell-cell adhesion and migration, encoded by the Cdh2 gene [13,14]. High N-cadherin expression is often associated with a reduction in cell proliferation [15,16] that, in osteoblasts, is mediated by the activation of Wnt3a signalling, which inhibits cyclin D1 expression [17,18]. Moreover, Zhao et al. demonstrated that N-Cadherin^High^ bone and marrow stromal progenitors are involved in the maintenance of the reserve (r)HSCs [19]. In cancer, N-cadherin has a different function according to the cellular context [16,20,21]. For example, in osteosarcoma, N-Cadherin works as a tumour suppressor [22], while in other cancers it promotes invasion [21]. In normal breast epithelial cells, N-Cadherin interacts with the fibroblast growth factor receptor (FGFR) and the Rho GTPase, activating the ERK signalling pathways and the expression of MMP9, eventually promoting cell motility [23]. In BrCa cells, N-Cadherin is mis-regulated and increases the Rho GTPase-induced cell motility. In addition, N-Cadherin provides BrCa cells with the capability to interact with the stroma and the endothelial cells, in order to migrate and become metastatic [24,25]. Moreover, a recent work demonstrated that N-Cadherin, in association with the connexin 43 expressed by the BrCa stem cells, mediates the communication between these latter and bone marrow cells [26]. Finally, although the data about the role of N-Cadherin in HSCs are conflicting [19,27,28,29], it is expressed by HSCs and promotes LT-HSC engraftment and quiescence in the bone marrow [29]. Nevertheless, the role of N-Cadherin in mediating SNO-induced BrCa cell dormancy and in HSCs mimicry is not fully understood and needs further investigation.

Based on this background, we hypothesised that N-Cadherin could play a role in BrCa dormancy and HSC-like stemness, cooperating with the Notch2 pathway in the process of dormancy and subsequent new tumour initiation ability. To demonstrate our hypothesis, we used mouse and human BrCa cell models in vitro and in vivo. We observed significant differences between human MDA and mouse 4T1 BrCa cells, unveiling a positive role of N-Cadherin in SNO-induced dormancy and HSC mimicry associated with the expression of Notch2 in MDA cells, and a negative role of N-Cadherin in 4T1 cell stemness and HSC mimicry, with a full independence of Notch2 and SNOs on the ability to inhibit cell proliferation.

## 2. Material and Methods

### 2.1. Materials

Dulbecco’s Modified Eagle Medium (DMEM) (cat: ECB7501L) penicillin–streptomycin (cat: ECB3001D), Dulbecco’s Phosphate Buffered Saline (DPBS) (cat: ECB4004L), Hanks’ Balanced Salt Solution (cat: ECB4007L) and disposable plastic were from Euroclone (Milan, Italy). Fetal bovine serum (FBS) (cat: 26140-079), Ethylene-Diamine-Tetra-acetic Acid (EDTA) (cat: 15576-028), TRIzol^®^ (Life Technologies, Carlsbad, CA, USA, cat: 15596018), N2 and B27 supplements (cat: 17502048, 17504044) and primers synthesis were from Invitrogen (Carlsbad, CA, USA). The cDNA Synthesis Kit (cat: K1622) was from ThermoFisher, Waltham, MA, USA. OneTaq^®^ Hot Start 2X Master Mix (cat: M0484S) and Luna^®^ Universal qPCR Master Mix (cat: M3003) were from New England BioLabs (Ipswich, MA, USA). Osteodec (cat: 05-03005E) and all reagents for histology were from Bio-Optica (Milano, Italy). SignalStain^®^ Boost IHC Detection Reagent (cat:8125S (anti-mouse) 8114S (anti-rabbit)) was from Cell Signaling (Danvers, MA, USA). All regents for magnetic-activated cell sorting (MACS) were from Miltenyi Biotec (Bergisch Gladbach, Germany). Mouse Cdh2 CRISPR/Cas9 plasmid (cat: KN503008) was from Origene (Rockville, MD, USA). Supplier, product code and dilution of primary and secondary antibodies used for the study are listed in Supplementary Table S1. Breast cancer tissue array was from BioChain^®^ (Newark, CA, USA) (cat: T8235721-5). ON-TARGET plus Human CDH2 siRNA (cat: FE5LHUMANXX0005) were purchased from Horizon Discovery, Waterbeach, UK. All other reagents, including Bovine Serum Albunin (BSA) (cat: A9418) and Clostridium histolyticum type IV collagenase (cat: C8051) were from Sigma Aldrich Co. (St. Louis, MO, USA).

### 2.2. Breast Cancer Cell Culture

Human MDA-MB-231 (MDA) and mouse 4T1 breast cancer cell lines, parental or transfected with turbo-GFP (MDA^GFP^ and 4T1^GFP^), were used for all experiments. Cells were cultured in high glucose Dulbecco’s Modified Eagle Medium (DMEM) with the addition of 1% glutamine, 1% penicillin–streptomycin and 10% FBS.

### 2.3. Genetically Modified 4T1 Breast Cancer Cells

Parental 4T1 cells were evaluated for N-cadherin expression by cytofluorimetry revealing, as expected, the presence of a 4T1 N-cadherin high subpopulation (Supplementary Figure S1a). N-Cadherin expression was then subjected to non-homology mediated CRISPR/Cas9 knockout by plasmids carrying two gRNAs targeting the N-Cadherin gene co-transfected with a linear donor containing genes encoding for the GFP tag and puromycin resistance (Supplementary Figure S1b). After stable transfection, the integration of the linear donor was confirmed in two 4T1 N-cadherin knock-out cell populations (4T1^Cdh2KO1-GFP^ and 4T1^Cdh2KO2-GFP^) using a primer pair specific for puromycin, confirming the success of the transfection protocol (Supplementary Figure S1c). Non-transfected 4T1 cells were included in the analyses as control. Cytofluorimetric analyses further confirmed the absence of N-Cadherin expression in the GFP-positive cells (knock-out cells) (Supplementary Figure S1d–g). After validation of transfection, 4T1^Cdh2KO1-GFP^ and 4T1^Cdh2KO2-GFP^ were sorted by FACS using the GFP tag. Then, to generate a rescued model, the FACS-sorted 4T1^Cdh2KO1-GFP^ and 4T1^Cdh2KO2-GFP^ were stably transfected with the mouse Cdh2-turboGFP expression vector (4T1^Cdh2Res1-GFP^ and 4T1^Cdh2Res2-GFP^), which restored N-Cadherin expression (Supplementary Figure S1h). Cells transfected with the empty-turboGFP vector (4T1^Cdh2KO1-GFP^ and 4T1^Cdh2KO2-GFP^) were used as control. Finally, the 4T1^Cdh2Res2-GFP^ that showed a non-stable Cdh2 over-expression, and the relative control knock-out cells (4T1^Cdh2KO2-GFP^) were excluded from the analysis (Supplementary Figure S1i). On the contrary, the 4T1^Cdh2KO1-GFP^ and the 4T1^Cdh2Res1-GFP^ were used for the experiments and labelled 4T1^Cdh2KO-GFP^ and 4T1^Cdh2Res-GFP^.

### 2.4. Mouse Primary Osteoblast Cell Isolation

Mouse primary osteoblasts were isolated from the calvariae of 8-day-old CD1 mice using three-step enzymatic digestion with a solution containing 25 mg/mL of porcine trypsin and 1 mg/mL of Clostridium histolyticum type IV collagenase in Hanks’ Balanced Salt Solution. The supernatant from the first digestion, containing mainly fibroblasts, was discarded, while those from the second and the third digestions, enriched in primary murine osteoblasts, were centrifuged at 300× *g* for 8 min and the cells were then cultured in high glucose DMEM supplemented with 1% glutamine, 1% penicillin–streptomycin and 10% FBS. At confluence, cells were trypsinised and plated according to the experimental protocol.

### 2.5. Magnetic-Activated Cell Sorting (MACS)

MDA and 4T1 parental or genetically modified cells, and primary mouse osteoblasts were sorted using MACS. Cells were detached with sorting buffer containing DPBS, 5% BSA and 0.5 M of EDTA. Resuspended cells were incubated for 20 min at 4 °C using N-Cadherin or Notch2 biotinylated primary antibodies. Then, cells were incubated again in the same condition using streptavidin-conjugated magnetic microbeads and were eluted through the magnetic column to separate the antigen-depleted and antigen-enriched cell populations. The cells obtained from this procedure were used for RNA isolation, in vitro assays, and in vivo experiments. Supplier, product code and dilution of the primary and secondary antibodies used for the MACS are listed in Supplementary Table S2.

### 2.6. RNA Extraction, RNA Deep Sequencing (RNAdSeq) Analysis and Gene Expression

RNA was extracted using TRIzol^®^ according to the manufacturer’s instructions. The RNA quality was assessed using electrophoresis agarose gel and was quantified by Nanodrop^®^ using an absorbance of 260 nm wavelength. The RNA purity was assessed measuring the 260/280 nm ratio and 260/230 nm ratio for the protein and phenol presence respectively.

For RNA dSeq, 3 independent RNA preparations for Notch2^High^ and Notch2^Low^ MDA cells were precipitated in ethanol and sent to Omega Bioservice (Norcross, GA, USA) for the RNA dSeq analysis. The generated RNA dSeq datasets, containing the expression profile of 36,000 genes for each sample/condition, was interrogated to examine the expression of the CDH2 gene.

For conventional gene expression analyses, 1 µg of RNA was retro-transcribed using Revertaid First Strand cDNA Synthesis. Semiquantitative PCR was performed using OneTaq^®^ Hot Start 2X Master Mix, while real-time PCR was performed using Luna^®^ Universal qPCR Master. Primer sequences used to assess gene expression are listed in Supplementary Table S1. Full agarose gel pictures are shown in Figures S6–S8.

### 2.7. Flow Cytometry

Cells were detached with a sorting buffer containing DPBS, 5% BSA and 0.5 M of EDTA. Resuspended cells were incubated with primary antibodies against Notch2 and N-Cadherin for 1 h at 4 °C. Then, secondary incubation was performed using fluorochrome-conjugated secondary antibodies; then, cells were analysed by the FACS Melody^®^ (BD) and FlowJO software. Unmarked cells were used to set the laser for the fluorescence threshold.

For the analysis of cells sorted from genetically modified 4T1 (4T1^Cdh2KO-GFP^ and 4T1^Cdh2Res-GFP^), the cell gaiting was performed using the GFP fluorescence. Supplier, product code and dilution of the primary and secondary antibodies used for the analyses are listed in Supplementary Table S1.

### 2.8. Animals

For the in vivo experiment, 4-week-old CD1 or Balb-C nude/nude (nu/nu) female mice were purchased from Charles River (Écully, France). Procedures involving animals and their care were conducted in conformity with national and international laws and policies (European Economic Community Council Directive 86/609, OJ L 358, 1, 12 December 1987; Italian Legislative Decree 116/92, Gazzetta Ufficiale della Repubblica Italiana no. 40, 18 February 1992; National Institutes of Health Guide for the Care and Use of Laboratory Animals, National Institutes of Health, 8th edition, 2011). The procedures were approved by the Institutional Ethical Review Board of the University of L’Aquila and by the Ministry of Health (Authorizations n° 270/2018-PR and 1151/2020-PR). The study was conducted according to the Animal Research Reporting In Vivo Experiments (ARRIVE) requirements.

### 2.9. Intratibial Injection of N-Cadherin^High^ and N-Cadherin^Low^ MDA and 4T1 Cells

Human MDA and mouse 4T1 BrCa cells were injected into the left tibia of 4-week-old female Balb/c nu/nu immunocompromised (for human cells) or Balb/c immunocompetent (for mouse cells) mice (1 × 10^4^ cells/0.01 mL PBS) anesthetized with intraperitoneal injection of 80 mg/kg of ketamine and 10 mg/kg of xylazine. Animals were monitored daily for body weight, food intake, behaviour, and survival.

To follow the progression of osteolytic lesions, mice were subjected to weekly X-ray analysis (X-ray parameters: peak kilovoltage [kVp] = 36 kV for 10 s) using a Cabinet X-ray system (Faxitron model no. 43855A; Faxitron X-Ray Corp., Buffalo Grove, IL, USA). At the end of the experiment, mice were subjected to final X-ray analysis and then sacrificed to perform anatomical dissection for the evaluation of bone and visceral metastases.

### 2.10. Limiting Dilution Assay (LDA)

Different dilutions (50,000 to 100 cells/mice) of human MDA cells MACS-sorted into N-Cadherin^High^ and N-Cadherin^Low^ subpopulations were subcutaneously injected in Balb/c nu/nu immunocompromised female mice anesthetized with intraperitoneal injection of 80 mg/kg of ketamine and 10 mg/kg of xylazine. Animals were monitored daily for body weight, food intake, behaviour, and survival. The tumor incidence was evaluated after 4 weeks from the injection of the tumor cells. The stem cell frequency was estimated using the Extreme Limiting Dilution Assay (ELDA) [30] available at https://bioinf.wehi.edu.au/software/elda/ (accessed on 25 February 2022).

### 2.11. Micro-Computed Tomography (µCT) Analysis

Left tibias harvested from the tumour cell-injected mice were fixed in 4% formaldehyde for 48 h and then scanned by μCT SkyScan 1174. The scan was performed with a 9.80 μm resolution using the X-ray voltage of 50 kV. The Skyscan NRecon software was used to reconstruct the images using a modified Feldkamp algorithm. Three-dimensional (3D) analysis was carried out employing a marching cubes-type model with a rendered surface. The cortical bone parameters were calculated on 300 consecutive slides starting from 100 µm below the growth plate, where the osteolytic lesions were located. Pratt’s algorithm was adopted to take 2D measurements. Threshold values were applied for segmenting trabecular bone. Bone cortical variables were selected according to Bouxsein et al. [31].

### 2.12. Histology

Left tibias were decalcified for 48 h in Osteodec and then embedded in paraffin. Livers were fixed in 4% paraformaldehyde and embedded in paraffin. Microtome sectioning was used to obtain tissue slices of 5-μm thickness. Liver sections were stained with haematoxylin and eosin while tibia sections were also processed for immunohistochemistry or immunofluorescence staining.

### 2.13. Immunohistochemistry and Immunofluorescence

For immunohistochemistry, mouse tibia sections and human primary BrCa tissue arrays were deparaffined and incubated with 0.07 M citrate buffer (pH 6) for 30 min at 96 °C and for 10 min at room temperature. The blocking was made with 3% H_2_O_2_ and 5% BSA. Then samples were incubated overnight at 4 °C with primary antibodies against N-Cadherin or human pan-Cytokeratin AE1/AE3. The staining signals were revealed using the SignalStain^®^ Boost IHC Detection Reagent (HPR rabbit or mouse). Sections were counterstained using Gill’s No.3 haematoxylin for 10 s. Positive and negative controls were performed in parallel.

For immunofluorescence, tissue sections or fixed cells (4% paraformaldehyde) were incubated with primary antibodies against human pan-Cytokeratin AE1/AE3, N-Cadherin or Ki67, either singularly or in combinations. Primary antibody incubations were carried out at room temperature for 1 h, then overnight at 4 °C. Then, incubations with secondary antibodies conjugated with AlexaFluor 488 or 594 were performed for 1 h at room temperature. Nuc-Spot^®^ or DAPI were used to stain the nuclei. The supplier, product code and dilution of the primary and secondary antibodies used for the analyses are listed in Supplementary Table S2.

### 2.14. Histomorphometry

Endosteal niche colonization analysis was performed for counting the number of cytokeratin positive cells in proximity of the endosteum (4 mm^2^ in area, 50 μm away from the growth plate and 20 μm away from the endocortical surface) [12], and their distance from the endosteal surface was measured. For liver metastases, sections were evaluated for metastasis number/mm^2^ and for the percentage of metastasis area over total tissue area. Primary BrCa tissue arrays were analysed counting the number of N-Cadherin^+^ cells on total surface. All histomorphometric analyses were performed using the software Fiji^®^ by ImageJ (version 1.53).

### 2.15. Osteoblast/BrCa Cells Coculture Assay

Mouse primary calvarial osteoblasts were MACS-sorted into SNOs or NON-SNOs using anti-N-Cadherin-biotin antibody and Streptavidin-conjugated magnetic microbeads, as described above. For the knock-down experiment, MACS-sorted MDA cells were incubated with CDH2- or Scramble (SCR)-siRNA for 48 h before proceeding with the coculture. 7 × 10^4^–1 × 10^5^ sorted cells were seeded in 96-well plates and incubated overnight in a humidified CO_2_ incubator (5% CO_2_, 37 °C). The day after, the MDA^GFP^ or the 4T1^GFP^, MACS-sorted into N-Cadherin^High^ and N-Cadherin^Low^ or Notch2^High^ and Notch2^Low^, or the non-sorted 4T1^Cdh2-KO-GFP^ and 4T1^Cdh2-Res-GFP^ were seeded on SNO or NON-SNO monolayers as above. After 1 h, cultures were extensively washed and the number of GFP^+^ cells were counted using an Olympus IX inverted fluorescence microscope. The counting was repeated after 24, 48, 72 h. BrCa cell density in the cocultures were 1 × 10^3^ for MDA^GFP^ cells and 1 × 10^2^ for 4T1^GFP^ cells.

### 2.16. Primary and Secondary Mammosphere Formation Assay

Primary mammosphere assays were performed using 8 × 10^3^ suspended cells seeded in non-adhesive Petri dishes with serum-free DMEM, supplemented with 1% N2, 1% B27, 1% penicillin/streptomycin and 1% L-glutamine. They were incubated for 6 days in a humidified CO_2_ incubator. For secondary mammospheres, the primary mammospheres were disaggregated using trypsin to obtain single-cell suspensions and cultured again under the same conditions. Imaging for the analysis was performed using the SXView Software (version 2.2.0.172). Mammosphere volume was calculated using the formula:


V = 3/4πr^3^. (1)

### 2.17. TaqMan™ Array Mouse Stem Cell Pluripotency

Two micrograms of total RNA isolated from 4T1^Cdh2KO-GFP^ and the 4T1^Cdh2Res-GFP^ cells were retrotranscribed into cDNA using Revertaid First Strand cDNA Synthesis. Then 200 ng of cDNA per condition were loaded in the TaqMan™ Array Mouse Stem Cell Pluripotency plates (Applied Biosystem cat: 4414080) containing specific Fluorescein Amidite (FAM)-tagged probes for mRNAs involved in mouse stemness and pluripotency. Real-time PCR was performed using the TaqMan™ Fast Advanced Master Mix (Applied Biosystem cat: 4444556). Gene expression was normalized by mouse Gapdh and expressed as fold vs. the 4T1^Cdh2KO-GFP^. The real-time arrays were run in triplicate.

### 2.18. Statistical Analyses

Results are expressed as mean ± Standard Deviation (SD). Sample size is indicated in the figure legends. Groups’ comparisons were performed carrying out independent samples Student’s *t*-tests and non-linear regression, fitting with F-test when dealing with continuous parameters. Data from RNA dSeq were analysed using a false discovery rate (FDR)-adjusted *p*-value. To assess the distributional pattern of the BrCa cells in the bone marrow in relation to the endosteal surface we used a cumulative frequency distribution with Gaussian regression and the F-test. For the ELDA, the *p*-value was calculated using a Student’s *t*-tests with 95% of confidence [30]. The statistical methods are indicated in the figure legends and the *p* values are indicated in the figures.

## 3. Results

### 3.1. N-Cadherin and Notch2 Are Co-Expressed in MDA Cells

**Figure 1:**
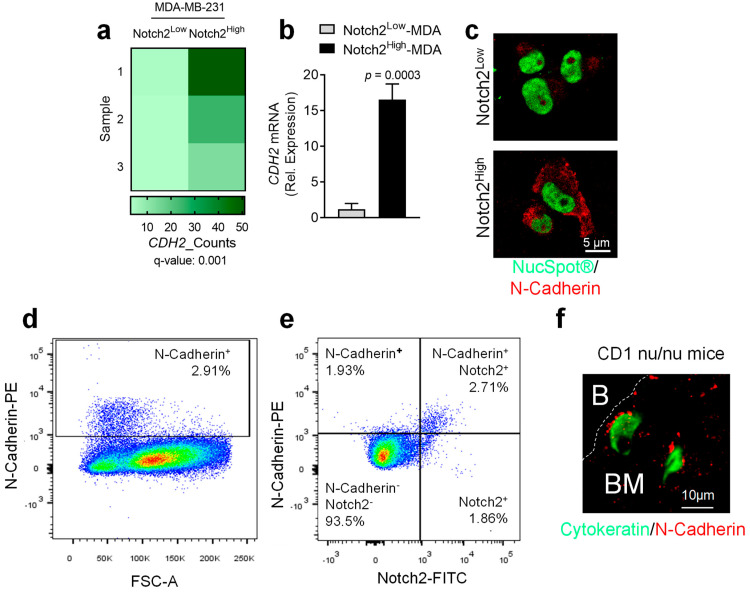
Analysis of N-Cadherin expression in Notch2^High^ MDA-MB-231 human BrCa cells. Total MDA-MB-231 (MDA) BrCa cells were MACS-sorted into Notch2^High^ and Notch2^Low^ subpopulations and the isolated RNA was subjected to RNA deep sequencing (RNA dSeq) analysis. (**a**) Heat map showing CDH2 expression in Notch2^High^ and Notch2^Low^ MDA cells. (**b**) Real-time RT-PCR performed using a specific primer pair for human CDH2. Human GAPDH was used to normalize gene expression. (**c**) Immunofluorescence staining performed on MACS-sorted Notch2^High^ and Notch2^Low^ MDA subpopulation to evaluate the expression of the N-Cadherin protein (red). NucSpot^®^ reagent was used to stain the nuclei (green). (**d**,**e**) Flow cytometry analysis of MDA cells stained with antibodies for N-Cadherin and Notch2. (**f**) Paraffin-embedded tibia sections harvested from CD1 nu/nu female mice intratibially injected with MDA cells for 4 weeks [12] were double-stained with antibodies for Cytokeratin (green) and N-Cadherin (red). B: bone; BM: bone marrow. (**c**,**e**) Scale bars are shown in the pictures. Data are the mean ± SD and pictures are representative of (**a**) three independent RNA dSeq datasets, (**b**–**d**) three independent cell cultures or (**e**) three mice. Statistical analysis: (**a**) FDR-adjusted *p*-value, (**b**) Student’s *t*-test.

Our previous data showed that the Notch2^High^ MDA BrCa cell line, which behaved as dormant cells in in vitro and in vivo BrCa dormancy models, also expressed N-Cadherin [12]. RNAdSeq confirmed that Notch2^High^ MDA cells were enriched in N-Cadherin mRNA (Figure 1a). Then, analysis carried out by real-time RT-PCR and immunofluorescence on independent samples of MACS-sorted Notch2^High^ and Notch2^Low^ MDA cells showed higher N-Cadherin expression in Notch2^High^ MDA cells (Figure 1b,c). Next, FACS analysis showed the presence of an N-Cadherin^High^ subgroup in the MDA cell population (Figure 1d). Moreover, an expanded analysis confirmed the presence of an MDA subpopulation co-expressing both Notch2 and N-Cadherin (Figure 1e). Finally, immunofluorescence for cytokeratin, performed on tibia sections of CD1 nu/nu female mice sacrificed after 4 weeks from intratibial injection of MDA cells, revealed the presence of single N-Cadherin/cytokeratin double-positive tumour cells close to the endosteal surface (Figure 1f).

These results suggested the presence of an MDA cell subpopulation co-expressing higher levels of both Notch2 and N-Cadherin that lodged in proximity of the endosteal area, prompting us to further investigate the role of N-Cadherin in the BrCa phenotype and dormancy.

### 3.2. Role of N-Cadherin in In Vivo Tumour Growth and Dormancy

**Figure 2:**
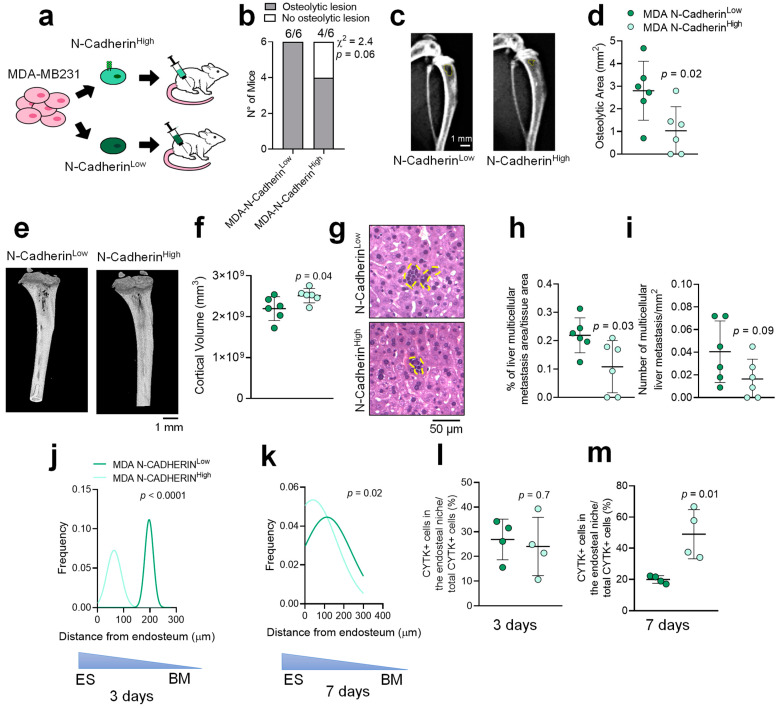
In vivo analysis of N-Cadherin^High^ in MDA BrCa cells. (**a**) Four-week old Balb-c nu/nu female mice were intratibially injected with 1 × 10^4^ MDA cells and MACS-sorted into N-Cadherin^High^ and N-Cadherin^Low^ subpopulations. (**b**) After 4 weeks, mice were sacrificed and tibial osteolytic lesion incidence and (**c**,**d**) area were analysed by X-rays while (**e**) µCT was used to visualize the 3D morphology and (**f**) to measure the cortical volume in the tibia of the injected-mice. (**g**) Paraffin-embedded livers isolated from MDA N-Cadherin^High^ and N-Cadherin^Low^-injected mice stained with haematoxylin and eosin to quantify (**h**) the multicellular liver metastases area over tissue area (%) and (**i**) the number of multicellular liver metastases over square millimetres by the ImageJ^®^ software. (**j**) Four-week old Balb-c nu/nu female mice were intratibially injected with 1 × 10^4^ MDA cells MACS-sorted into N-Cadherin^High^ and N-Cadherin^Low^ subpopulations and sacrificed 3- and (**k**) 7-days post-injection. (**l**) Paraffin-embedded tibias were harvested from the injected mice immuno-stained for cytokeratin to visualize the tumour cell in the bone tissue and measure the cell distribution in relation to the endosteum, and (**m**) the number of cells in the endosteal niche at the indicated time points. Data are the mean ± SD and pictures are representative of 4–6 mice per group. Statistical analysis: (**b**) χ square analysis, (**d**,**f**,**h**,**i**,**l**,**m**) Student’s *t*-test, (**j**,**k**) Gaussian curve regression fitting and F-test. Scale bars are shown in the pictures.

To address the relevance of N-Cadherin in the in vivo tumour growth and dormancy, MACS-sorted N-Cadherin^High^ and N-Cadherin^Low^ MDA cells were injected into the tibia medullary cavity of female Balb-c nu/nu mice (Figure 2a). After 4 weeks, X-rays and μCT analyses showed a trend of reduction in osteolytic lesion incidence (chi-square test; *p* = 0.06) (Figure 2b and Supplementary Figure S2a) along with a reduced osteolytic area (Figure 2c,d) in the tibias of N-Cadherin^High^ compared to N-Cadherin^Low^ cell-injected mice. Accordingly, μCT analysis revealed a higher cortical bone volume (Ct.V) in N-Cadherin^High^ cell-injected mice (Figure 2e,f). Interestingly, histopathological analyses revealed that the liver metastases were smaller in N-Cadherin^High^ compared to N-Cadherin^Low^ cell-injected mice (Figure 2g,h), with a trend of reduction in their number and incidence (Figure 2i and Supplementary Figure S2b). Lung metastases were instead undetectable.

Next, we analysed the endosteal niche colonization capability of Cadherin^High^ and N-Cadherin^Low^ MDA cells at 3- and 7-days post-injection, evaluating the tumour cell distribution in relation to their vicinity to the endosteal surface. Histomorphometric analysis demonstrated that the distance of cytokeratin^+^ MDA cells from the endosteal surface was lower in the N-Cadherin^High^ compared to the N-Cadherin^Low^ cell-injected mice at all the time points analysed (Figure 2j,k). In line with this observation, the number of MDA cells nearby the endosteal surface was higher in the N-Cadherin^High^ cell-injected mice after 7 days (Figure 2l,m).

Finally, we investigated if these features were shared by the mouse breast cancer cell line, 4T1. Of note, we did not observe significant differences in the in-bone tumour growth and endosteal niche colonization capability when we injected MACS-sorted N-Cadherin^High^ or N-Cadherin^Low^ 4T1 cells into the tibia medullary cavity of female Balb/C mice after 3 and 7 days (Supplementary Figure S2c–e). Longer term experiments were not possible due to the high aggressiveness of the 4T1 that grew fast in the tibia and quickly colonised the liver (Supplementary Figure S2f).

Altogether, these results demonstrated that the expression of N-Cadherin is associated with reduced MDA cell aggressiveness in the bone microenvironment and increased ability to home to the endosteal niche. Furthermore, these features were unique to the human MDA cells, not shared, at least in our experimental conditions, by the mouse 4T1 cells.

### 3.3. N-Cadherin Mediates BrCa Cell Adhesion onto SNOs In Vitro

**Figure 3:**
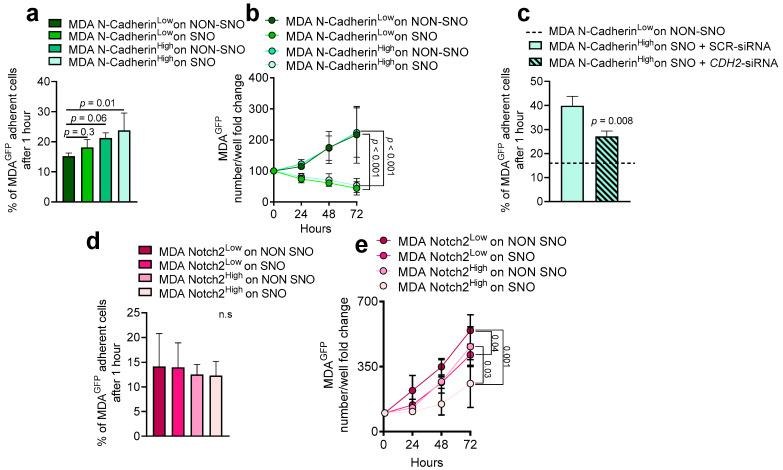
Role of N-Cadherin and Notch2 in MDA-SNO interaction in vitro. (**a**) N-Cadherin^High^ and N-Cadherin^Low^ MDA^GFP^ cells were seeded onto MACS-sorted SNOs and NON-SNOs and allowed to attach for 1 h at 37 °C, followed by extensive washing. Number of MDA^GFP^ cells was assessed after 1h of adhesion and (**b**) after 24–72 h of co-culture. (**c**) N-Cadherin^High^ and N-Cadherin^Low^ MDA^GFP^ cells, treated with siRNA against the N-Cadherin (CDH2-siRNA) or scrambled (SCR-siRNA), were seeded onto MACS-sorted SNOs and NON-SNOs. The number of MDA^GFP^ cells was assessed after 1h of adhesion. (**d**) Notch2^High^ and Notch2^Low^ MDA^GFP^ cells were seeded onto MACS-sorted SNOs and NON-SNOs and allowed to attach for 1 h at 37 °C, followed by extensive washing. The number of MDA^GFP^ cells was assessed after 1h of adhesion and (**e**) after 24–72 h of co-culture. In (**b**,**d**) cell number per well was normalized for time 0 (number of cells after 1 h of adhesion). Data are the mean ± SD of 4 independent cell preparations. Statistical analysis: (**a**,**c**,**d**) Student’s *t*-test, (**b**,**e**) Non-linear regression fitting and F-test.

To establish the role of N-Cadherin in the SNO-mediated tumour cell dormancy, MACS-sorted N-Cadherin^High^ and N-Cadherin^Low^ MDA^GFP^ cells were seeded onto sorted SNOs or NON-SNOs as previously described [12]. N-Cadherin^High^ MDA^GFP^ cell adhesion, measured after 1 h from plating, was significantly higher when they were plated on SNOs compared to all other conditions tested (Figure 3a). In a time-course of 24–72 h, the number of MDA^GFP^ cells was lower in MDA-SNO compared to MDA-NON-SNO co-cultures, regardless of the N-Cadherin status (Figure 3b), suggesting that the expression of N-Cadherin does not affect the inhibition of MDA cell proliferation induced by SNOs [12]. In line with this observation, the N-Cadherin knock-down by a specific siRNA reduced the N-Cadherin^High^ MDA^GFP^ cell adhesion to SNOs (Figure 3c) without affecting the SNO-dependent inhibition of MDA proliferation (Supplementary Figure S3a).

Interestingly, N-Cadherin^High^ MDA cells showed higher Notch2 transcriptional expression when compared with the N-Cadherin^Low^ counterpart (Supplementary Figure S3b), and FACS analysis revealed that 58.4% of the N-Cadherin^High^ MDA cells were also Notch2^High^ (Figure 1e). We also co-cultured MACS-sorted Notch2^High^ and Notch2^Low^ MDA^GFP^ cells with SNOs or NON-SNOs. Although cell adhesion measured after 1 h from plating was not statistically different in Notch2^High^ and Notch2^Low^ MDA^GFP^ co-cultured with SNOs and NON-SNOs (Figure 3d), during the subsequent time-course the number of Notch2^High^ MDA^GFP^ cells increased less when plated on SNOs compared to the co-plating with NON-SNOs (Figure 3e). Interestingly, co-cultures between Notch2^High^ MDA^GFP^ cells and NON-SNOs or Notch2^Low^ MDA^GFP^ cells and SNOs impaired tumour cell proliferation only partially, suggesting that both players should be present to induce the maximum inhibitory effect (Figure 3e).

Next, we tested the role of N-Cadherin in SNO-induced BrCa dormancy using the mouse 4T1 cells. To this purpose, we generated genetically modified 4T1 cells using the CRISPR/Cas9 technology. Briefly, N-Cadherin expression was firstly abolished (4T1^Cdh2-KO^) and subsequentially rescued (4T1^Cdh2-Res^) (Supplementary Figure S1).

**Figure 4:**
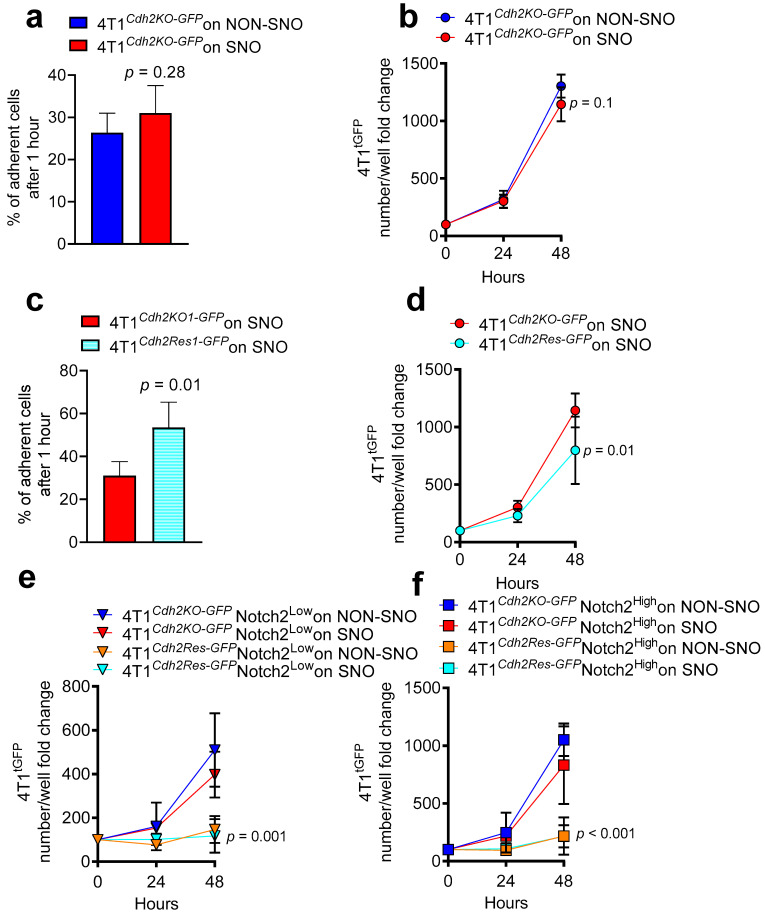
Role of N-Cadherin and Notch2 in 4T1-SNO interaction in vitro. (**a**) 4T1^GFP^ cells knocked-out for N-Cadherin expression (4T1^Cdh2-KO-GFP^) were seeded onto MACS-sorted SNOs and NON-SNOs and allowed to attach for 1 h at 37 °C, followed by extensive washing. The number of 4T1^GFP^ cells was assessed after 1h of adhesion and (**b**) after 24–48 h of co-culture. (**c**) 4T1^GFP^ cells in which the N-Cadherin expression was rescued (4T1^Cdh2-Res-GFP^) were seeded onto MACS-sorted SNOs and allowed to attach for 1 h at 37 °C, followed by extensive washing. The number of 4T1^GFP^ cells was assessed after 1h of adhesion and (**d**) after 24–48 h of co-culture. (**e**) Notch2^High^ and (**f**) Notch2^Low^ 4T1^Cdh2-KO-GFP^ and 4T1^Cdh2-Res-GFP^ cells were seeded onto MACS-sorted SNOs and NON-SNOs cultured for 24 h and allowed to attach for 1 h at 37 °C, followed by extensive washing. Number of 4T1^GFP^ cells was assessed after 24–48 h of co-culture. (**a**,**d**–**f**) Cell number per well was normalized for time 0 (number of cells after 1h of adhesion). Data are the mean ± SD of 4–5 independent cell preparations. Statistical analysis: (**a**,**c**) Student’s *t*-test, (**b**,**d**–**f**) non-linear regression fitting and F-test.

In our previous work, we demonstrated that SNOs induced cellular dormancy in parental unsorted 4T1 cells [12]. Here, we showed no difference in adhesion and number of 4T1^Cdh2-KO-GFP^ cells co-cultured with SNOs or NON-SNOs for 48 h (Figure 4a,b). Interestingly, the number of 4T1^Cdh2-Res-GFP^ cells plated onto SNOs was higher after 1h of co-plating (Figure 4c), but their number increased less in the subsequent 48 h compared to 4T1^Cdh2-KO-GFP^ (Figure 4d).

Of note, unlike MDA cells, in the 4T1 cells we observed that the level of Notch2 expression was independent of N-Cadherin expression. In fact, no significant differences were found in Notch2 expression between 4T1^Cdh2-KO-GFP^ and 4T1^Cdh2-Res-GFP^ cells and between N-Cadherin^High^ and N-Cadherin^Low^ MACS-sorted 4T1 cells (Supplementary Figure S4a,b). In agreement with this observation, when 4T1^Cdh2-KO-GFP^ and 4T1^Cdh2-Res-GFP^ cells MACS-sorted into Notch2^High^ and Notch2^Low^ were co-plated with SNOs or NON-SNOs, we confirmed that the slower growth rate in the 4T1 cells was related to the presence of N-Cadherin and not to the Notch2 status. In fact, in all conditions tested, 4T1^Cdh2Res-GFP^-Notch2^High^ and 4T1^Cdh2Res-GFP^-Notch2^Low^ cultures showed the lowest number of cells per plate when compared to 4T1^Cdh2KO-GFP^-Notch2^High^ and 4T1^Cdh2KO-GFP^-Notch2^Low^ cells plated onto both SNOs and NON-SNOs for 48 h (Figure 4e,f). These results suggest that the axis Notch2/N-Cadherin is not involved in the SNO-mediated cell dormancy, while N-Cadherin strongly reduces the proliferation ability of 4T1 cells both on SNOs and NON-SNOs.

Taken together these data indicated that N-Cadherin plays a role in MDA and 4T1 cell adhesion especially onto SNOs, while Notch2 is pivotal in SNO-induced dormancy in human MDA cells, but not in mouse 4T1 cells.

### 3.4. The Role of N-Cadherin in HSC Mimicry and Cancer Stem Cell-like Phenotype

**Figure 5:**
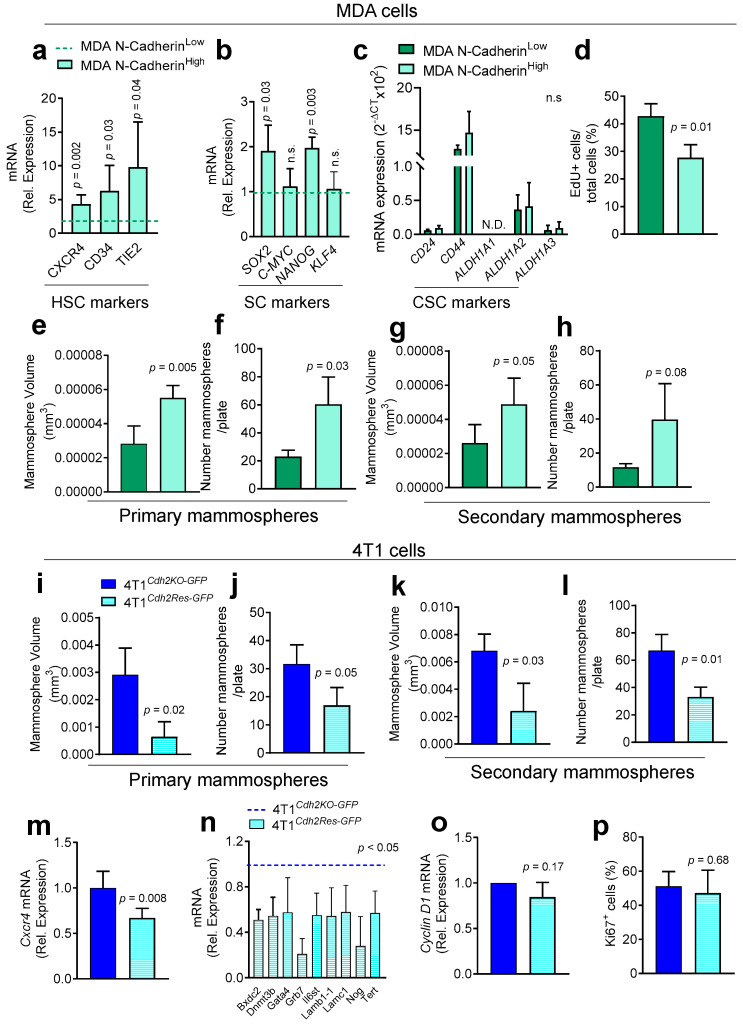
Effect of N-Cadherin expression on HSC mimicry and stemness of MDA and 4T1 BrCa cells. MDA cells were sorted into N-Cadherin^High^ and N-Cadherin^Low^ subpopulations by MACS. Real-time RT-PCR was used to assess the expression of the indicated (**a**) HSCs, (**b**) Stem Cell (SC) and (**c**) Cancer Stem Cell (CSC) markers. Human GAPDH was used to normalize gene expression. (**d**) 5-ethynyl-2′-deoxyuridine (EdU) assay was used to assess cell proliferation. (**e**) Size and (**f**) number of primary mammospheres obtained from MACS-sorted N-Cadherin^High^ and N-Cadherin^Low^ MDA cells. (**g**) Size and (**h**) number of secondary mammospheres obtained after trypsinization and re-plating single cells harvested from the primary MDA mammospheres. (**i**) Size and (**j**) number of primary mammospheres obtained from 4T1^Cdh2-KO^ and 4T1^Cdh2-Res^ GFP cells. (**k**) Size and (**l**) number of secondary mammospheres obtained after trypsinization and re-plating of single cells harvested from the primary 4T1 mammospheres. (**m**) Expression of the HSC marker Cxcr4 in 4T1^Cdh2-KO^ and 4T1^Cdh2-Res^ cells. Gene expression was normalised by mouse Gapdh. (**n**) Real-time array to assess the expression of the canonical mouse stem genes in 4T1^Cdh2-KO^ and 4T1^Cdh2-Res^ cells. The graph shows the genes that were found significantly modulated. The complete list is reported in Supplementary Table S1. (**o**) Cell proliferation assessed in the 4T1^Cdh2-KO^ and 4T1^Cdh2-Res^ cells by real-time RT-PCR to evaluate the expression of Cyclin D1 and (**p**) by immunofluorescence using a specific antibody for the Ki67. Data are the mean ± SD of 3–5 independent cell preparations. Statistical analysis: Student’s *t*-test.

We previously demonstrated that the dormant Notch2^High^ MDA cells showed HSC mimicry, along with a cancer stem cell-like phenotype and reduced 2D cell proliferation, when compared with the Notch2^low^ counterpart [12]. To evaluate whether these features were shared by the N-Cadherin^High^ BrCa cells, we first investigated whether MACS-sorted N-Cadherin^High^ MDA cells showed an HSC-like gene signature. Our analysis demonstrated that they expressed higher mRNA levels of the HSC markers, CD34, TEK Receptor Tyrosine Kinase (TIE2), and C-X-C Motif Chemokine Receptor 4 (CXCR4) compared to N-Cadherin^Low^ MDA cells (Figure 5a), along with a higher expression of the stem cell related genes SOX2 and NAGOG (Figure 5b). Moreover, we confirmed the presence of a canonical cancer stem cell signature independent from N-Cadherin expression. Indeed, both N-Cadherin^High^ and N-Cadherin^Low^ MDA cells expressed high CD44, medium ALDH and low CD24 mRNA (Figure 5c and Supplementary Figure S5a). On the contrary, a lower expression of the cell proliferation marker EdU was found in N-Cadherin^High^ compared to N-Cadherin^Low^ MDA cells (Figure 5d). Of note, N-Cadherin^High^ cells were able to initiate more primary (Figure 5e,f) and secondary (Figure 5g,h) mammospheres. Moreover, primary mammospheres from the N-Cadherin^High^ MDA cells were larger (Figure 5e) and more numerous (Figure 5f) than mammospheres generated by N-Cadherin^Low^ MDA cells. Secondary mammospheres were larger in the N-Cadherin^High^ compared to the N-Cadherin^Low^ MDA cells (Figure 5g), while their number was very variable and showed only a trend to increase (Figure 5h). The cancer stem cell-like phenotype was further confirmed employing an in vivo Limiting Dilution Assays (LDA) that revealed a higher stem cell frequency in the N-Cadherin^High^ compared to the N-Cadherin^Low^ MDA cells subpopulation (Table 1).

Interestingly, the analysis of the epithelial-to-mesenchymal transition (EMT) markers revealed a higher expression of E-Cadherin in the N-Cadherin^High^ vs. N-Cadherin^Low^ MDA cells (Supplementary Figure S5b).

Next, the phenotypic comparison between MDA and 4T1 cells revealed a different impact of N-Cadherin on cellular stemness. In fact, unlike MDA cells, both 4T1^Cdh2-Res-GFP^ and 4T1 N-Cadherin^High^ showed a lower ability to initiate both primary (Figure 5i–j and Supplementary Figure S5c,d) and secondary (Figure 5k–l and Supplementary Figure S5e,f) mammospheres. Moreover, primary and secondary mammospheres from the 4T1^Cdh2-Res-GFP^ and 4T1 N-Cadherin^High^ cells were smaller (Figure 5i,k and Supplementary Figure S5c,e) and less numerous (Figure 5j,l and Supplementary Figure S5d,f) than mammospheres generated by the 4T1^Cdh2KO-GFP^ and 4T1 N-Cadherin^Low^ cells. In line with this observation, Cxcr4 expression was lower in 4T1^Cdh2Res-GFP^ compared to 4T1^Cdh2KO-GFP^ cells (Figure 5m) while it was unremarkable in the N-Cadherin^High^ compared to N-Cadherin^Low^ 4T1 cells (Supplementary Figure S5g). Further investigation by TaqMan™ Array Mouse Stem Cell Pluripotency revealed a significantly lower expression of genes involved in mouse stemness, in 4T1^Cdh2Res-GFP^ compared to 4T1^Cdh2KO-GFP^ cells (Figure 5n and Supplementary Table S3). Finally, cell proliferation was unchanged in both 4T1^Cdh2-Res-GFP^ and 4T1 N-Cadherin^High^ compared to the 4T1^Cdh2KO-GFP^ and 4T1 N-Cadherin^Low^ cells (Figure 5o,p and Supplementary Figure S5h). Of note, the analysis of the EMT markers revealed a lower expression of E-Cadherin in 4T1^Cdh2Res-GFP^ vs. 4T1^Cdh2KO-GFP^ and a reduced vimentin expression in 4T1 N-Cadherin^High^ compared to 4T1 N-Cadherin^Low^ cells (Supplementary Figure S5i–j).

Taken together these results showed that, unlike 4T1, N-Cadherin^High^ MDA cells display an HSC-like gene signature and a cancer stem cell-like phenotype similar to the Notch2^High^ MDA cells. Moreover, the role of N-Cadherin in mouse 4T1 cancer cell stemness differs from its role in human MDA cell stemness.

### 3.5. N-Cadherin Status in Human Primary BrCa and Correlation with Survival

**Figure 6:**
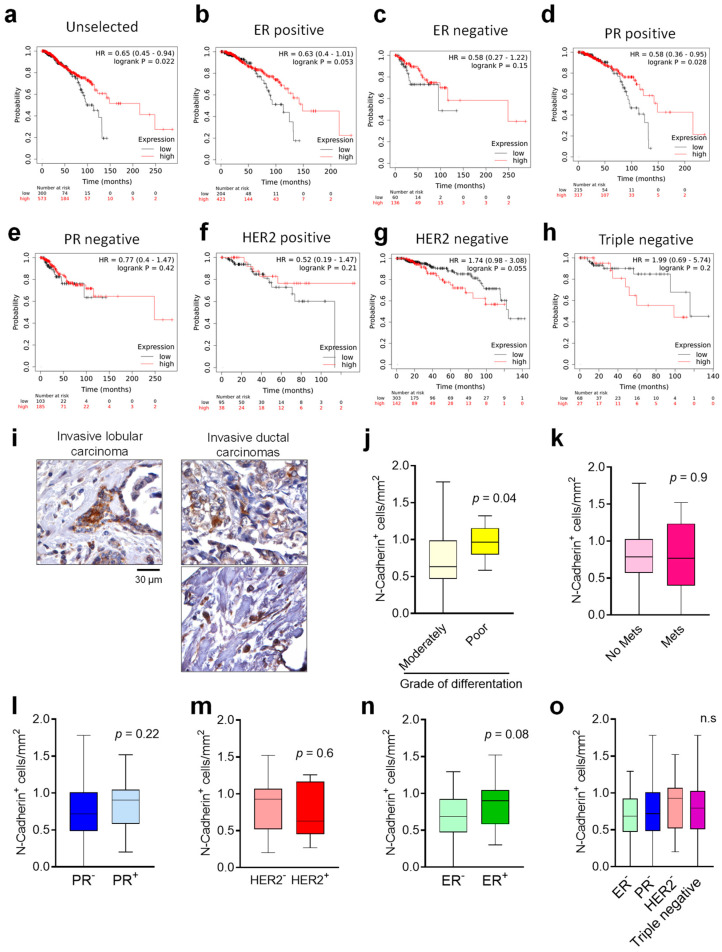
N-Cadherin expression in primary human breast cancers and correlation with survival. (**a**) Kaplan–Meier plots on 1229 public proteomics from primary breast cancers to correlate N-Cadherin protein expression with patient survival in unselected populations of 300 N-Cadherin^Low^ and 573 N-Cadherin^High^ samples, (**b**) in oestrogen receptor (ER)-positive of 204 N-Cadherin^Low^ and 423 N-Cadherin^High^ samples, (**c**) in ER-negative populations of 60 N-Cadherin^Low^ and 136 N-Cadherin^High^ samples, (**d**) in progesterone receptor (PR)-positive of 215 N-Cadherin^Low^ and 317 N-Cadherin^High^ and (**e**) progesterone receptor (PR)-negative populations of 103 N-Cadherin^Low^ and 185 N-Cadherin^High^ samples, (**f**) in HER2-positive populations of 95 N-Cadherin^Low^ and 38 N-Cadherin^High^ samples, (**g**) in HER2-negative populations of 303 N-Cadherin^Low^ and 142 N-Cadherin^High^ samples and (**h**) in triple negative populations of 68 N-Cadherin^Low^ and 27 N-Cadherin^High^ samples plotted against time (KMPlot^®^). (**i**) Breast cancer tissue array containing 64 primary breast cancer samples was stained for N-Cadherin by immunohistochemistry. The number of the N-Cadherin positive cells was quantified, and the results were stratified according to (**j**) grade of differentiation, (**k**) presence of distal metastases, the expression of (**l**) PR, (**m**) HER2 or (**n**) ER receptors and in (**o**) triple negative primary tumours. Pictures are representative and data in (**j**–**o**) are the mean ± SD of at least 9 primary tumours per condition. Statistical analysis: (**a**,**h**) log-rank test; (**j**–**o**) Student’s *t*-test.

The KMplot^®^, containing protein expression data and survival information from four independent cohorts of 1193 BrCa patients, was used to test the correlation between N-Cadherin protein expression and survival. The analysis revealed the presence of a positive correlation between the N-Cadherin and the overall survival when we analysed the whole dataset (Figure 6a). Furthermore, when data were stratified for oestrogen (ER), progesteron (PR) receptor, Human Epidermal growth factor Receptor 2 (HER2) or triple negative subtypes, we confirmed a significant and positive correlation between high N-cadherin level and overall survival in patients with either ER- or PR-positive breast cancers (Figure 6b,d). On the contrary, no significant correlations were found in the survival of patients with either ER- or PR-negative, HER2 positive and negative, or triple negative breast cancers (Figure 6c,e,f–h).

Finally, we investigated the expression of N-Cadherin in a BrCa tissue array containing 64 different samples of primary tumours. We observed that N-Cadherin positive cells represented a small subpopulation (0.76 ± 0.3 cell/mm^2^) (Figure 6i and Supplementary Table S4). Moreover, histopathological analysis revealed that the number of N-Cadherin positive cells was higher in poorly differentiated primary BrCa (Figure 6j). In contrast, the number of N-cadherin positive cells was unchanged when we stratified the tumours according to the presence of distant metastasis (Figure 6k) and PR and HER2 status (Figure 6l,m), while, when we stratified our samples for the ER status, a trend of increase in the N-cadherin-positive cells (*p* = 0.08) was found in the ER-positive compared to the ER-negative primary breast cancers (Figure 6n). Moreover, no changes in the number of N-Cadherin positive cells was found when we compared the ER, PR and HER2 single negative with triple negative specimens (Figure 6o).

## 4. Discussion

Tumour cell dormancy is an intricate mechanism involving different molecular pathways and cell-cell interactions in accordance with the type of cancer and the microenvironmental signalling [32]. In our previous work we demonstrated that dormant BrCa cells interact with a specific osteoblast subpopulation, known as spindle-shaped N-Cadherin^High^ osteoblasts (SNOs), remaining cell cycle arrested due to the Notch2 pathway. Moreover, we demonstrated that dormant BrCa cells compete with HSCs for bone marrow engraftment and endosteal niche colonization thanks to their HSC mimicry features [12]. In this work, we added a new piece in this complex puzzle showing a potential role of N-Cadherin in the SNO-mediated BrCa cell dormancy and cellular stemness.

The role of N-Cadherin in tumour biology is very complex, and varies according to the cellular context and the type of tumour [21]. N-cadherin has been reported to induce or suppress tumour development and spreading according to the type of cancer [22,24,25,26]. In the BrCa context, the role of N-Cadherin is still poorly understood, probably because its function is tightly related to cellular and microenvironmental conditions. Data in the literature demonstrate that the expression of N-Cadherin increases BrCa cell adhesion to the stroma and stimulates motility, enhancing metastatic spread [15,25,26,33]. In contrast, a recent paper demonstrated that N-Cadherin drives human BrCa dormancy in the bone marrow in association with connexin 43 [26]. Moreover, in line with our findings, the author showed that N-Cadherin was expressed by the stem compartment of BrCa cells, contributing to the maintenance of the cellular dormancy [26].

We found the expression of N-Cadherin in the dormant Notch2^High^ MDA BrCa cells, confirming the possible association between N-Cadherin expression and BrCa cellular dormancy. In line with this, MDA cells expressing high level of N-Cadherin showed a lower aggressiveness in the bone microenvironment associated with a lower incidence of osteolytic lesions alongside an increased endosteal niche engraftment, indicating that the N-Cadherin signalling prompted the tumour cells to acquire a dormant-like phenotype. The ability to lodge in proximity of the endosteal niche led us to assume that they were able to interact with the SNOs. Our assumption was confirmed in vitro by experiments showing that MDA cells expressing N-Cadherin were able to interact with SNOs and that the presence of N-Cadherin increased the ability of tumour cells to adhere to SNOs. This observation was further confirmed by the fact that the knock-down of the N-Cadherin expression in MDA cells reduced their ability to adhere to SNOs. Of note, unlike Notch2^High^ cells, the proliferation of the N-Cadherin^High^ MDA cells in the presence of SNOs was unremarkable, suggesting that N-Cadherin mediates the adhesion of BrCa cells to SNOs, while Notch2 mediates the inhibition of SNO-induced cell proliferation. Accordingly, further analyses confirmed that only about 58% of N-Cadherin^High^ MDA expressed high levels of Notch2. Altogether these data prompted us to hypothesize that the homophilic N-Cadherin interaction between MDA cells and SNOs is used by tumour cells for engraftment to the endosteal niche, while only a small subpopulation co-expressing Notch2 also acquires the dormant phenotype.

As mentioned, the ability to colonize the endosteal niche and interact with SNOs is a typical feature of HSCs [10,19] shared also by the dormant Notch2^High^ MDA cells [12]. Intriguingly, although conflicting data about the role of N-cadherin in HSCs are described in literature [19,27,28,29], Arai et al. demonstrated that N-Cadherin is expressed by HSCs and that its overexpression promotes HSC quiescence [29]. In agreement with this observation, our data showed that the N-Cadherin expression was associated with the acquisition of a bone marrow-specific cancer stem cell phenotype and HSC like-signature in MDA cells. In fact, we found a higher expression of the canonical HSC markers CXCR4, TIE-2 and CD34 and of the stem cell related markers SOX2 and NANOG in the N-Cadherin^High^ MDA cells along with a lower cell proliferation rate and a higher ability to form primary and secondary mammospheres. Moreover, in vivo LDA showed a higher stem cell frequency in the N-Cadherin^High^ MDA cells, further confirming their ability to initiate a new cancer. Interestingly, the analysis of canonical cancer stem cell markers revealed that both N-Cadherin^High^ and N-Cadherin^Low^ MDA cells display the typical cancer stem cell phenotype CD44^High^/CD24^Low^/ALDH^+^ [34]. These findings suggest that N-Cadherin expression identifies a specific subpopulation, expressing HSC- and stem cell-related markers, within the canonical CD44^High^/CD24^Low^/ALDH^+^ cancer stem cell population. These data were in line with the ability of the N-Cadherin^High^ to colonize the endosteal niche resulting in lower aggressiveness in the bone/bone marrow microenvironment and in the concept that dormant cells should have stem features to initiate a new tumour after their reactivation [6,7].

We also performed our experiments using the mouse BrCa cell line 4T1, and were able to establish syngeneic interactions with mouse bone cells in vitro and in vivo. We demonstrated that N-Cadherin is required in 4T1 cells for the interaction with SNOs in vitro. Indeed, the lack of N-Cadherin reverted the impairment of the 4T1 cell proliferation induced by SNOs. On the other hand, the re-expression of the N-Cadherin enhanced the adhesion of 4T1 cells onto SNOs, slowing down their proliferation. On the contrary, in vivo analysis of 4T1 revealed no differences associated with N-Cadherin expression. This was probably because 4T1 are characterised by a strong aggressiveness and extremely high proliferation rate in the bone microenvironment. Interestingly, in 4T1 cells there was no co-expression of N-Cadherin and Notch2, indicating that different signalling pathways could be involved in mouse BrCa cellular dormancy. This was also confirmed by the observation that, unlike MDA cells, 4T1 cells expressing a high level of Notch2 showed a higher proliferation rate when co-cultured with SNOs.

Surprisingly, when we analysed the stem features of 4T1 cells expressing N-Cadherin we found an opposite effect compared to the MDA cells. Indeed, we observed that high N-Cadherin expression was associated with a lower expression of the HSC marker Cxcr4 and the mouse stem related genes Bxdc2, Dnmt3b, Gata4, Grb7, Il6st, Lamb1-1, Lamc1, Nog and Tert, along with a lower ability to form primary and secondary mammospheres. Other HSC markers including Tie-2 and Sca-1 were not expressed by 4T1 cells in our experimental conditions. These results were confirmed using both genetic manipulation of the N-Cadherin and cell sorting for N-Cadherin status.

Finally, according to the fact that there are no reliable markers to predict tumour dormancy in clinical practice [35], we investigated whether N-Cadherin could be useful to this scope. The analyses carried out using public datasets demonstrated that N-Cadherin protein expression in the primary tumour was correlated with a better prognosis in an unselected BrCa patients’ cohort. In line with this, the analyses of a primary BrCa tissues array demonstrated a trend of increase in the number of N-Cadherin positive cells in ER positive tumours, known to be less malignant, suggesting that N-Cadherin expression could be associated with less aggressive human primary tumours. However, we also found a higher number of N-Cadherin-positive cells in primary tumours classified as less differentiated, which are known to be more aggressive [36]. This is in line with a recent publication showing that N-Cadherin expression in patients with ductal carcinoma in situ is predictive of synchronous invasion [37]. Another explanation of this conflicting result could be represented by the fact that less differentiated tumour cells usually show a stem-like phenotype. In confirmation, according to our and other’s [12,26,38] results, N-Cadherin is likely to be expressed mainly in the stem cellular compartment. A limitation of this study is that most of the tumours analysed were ductal carcinomas, and that the absence of more differentiated tumours in our BrCa tissue array did not allow a complete correlation analysis between type and differentiation grade of primary tumours and N-Cadherin expression. Moreover, even if our data were generated using triple negative BrCa cell lines, we did not find association between N-Cadherin expression and the overall survival in patients harboring triple negative breast cancers. Similar results were found when we analysed the number of N-Cadherin positive cells in triple negative specimens present in our BrCa tissues array. This could be partially explained by the fact that the number of patients derived from the public dataset carrying a triple negative tumor was relatively low (68 N-Cadherin^Low^ and 27 N-Cadherin^High^ samples) and the absence of triple positive specimens in our BrCa tissue array forced us to compare the triple negative samples only with the ER, PR and HER2 single negative tumors.

For this reason, further larger studies are needed to clarify the possible role of N-Cadherin as an early dormancy marker in BrCa patients.

## 5. Conclusions

In conclusion, in this work we defined the role of N-Cadherin in BrCa dormancy and stemness, highlighting the differences between human and mouse cell lines. Overall, we can conclude that N-Cadherin could play a role in the induction and maintenance of tumour cell dormancy, in cooperation with Notch2. Moreover, we demonstrated that N-Cadherin is mainly involved in the anchorage of tumour cells to the endosteal niche, rather than in the inhibition of cell proliferation. Therefore, we can speculate that targeting N-Cadherin could be a potential co-adjuvant therapy to be administered in combination with anti-tumoral drugs to prevent the engraftment and the dormancy of BrCa cells lodged in the endosteal niche.

## Figures and Tables

**Table 1:** Stem cell frequency in N-Cadherin^High^ and N-Cadherin^Low^ MDA tumors.

Cell Models	Cell Number			Stem Cell Frequency (ELDA)	*p*-Value (vs. N-Cadherin^Low^)
	50,000	10,000	100		
N-Cadherin^Low^-MDA	5/5 (100%)	4/5 (80%)	0/5 (0%)	1/6338	-
N-Cadherin^High^-MDA	5/5 (100%)	5/5 (100%)	2/5 (40%)	1/196	0.002

## Data Availability

Data are available from the authors upon request to the corresponding author.

## Supplementary Materials

The following are available online at https://www.mdpi.com/article/10.3390/cancers14051317/s1, Figure S1: Generation of genetically modified 4T1 cells, Figure S2: In vivo analysis of MDA- and 4T1-N-Cadherin^High^ BrCa cells, Figure S3: Effect of N-Cadherin down regulation on proliferation to SNOs and NON-SNOs and Notch2/N-Cadherin co-expression analysis in MDA cells, Figure S4: Notch2 and N-Cadherin co-expression analysis in 4T1 cells, Figure S5: Effect of N-Cadherin expression on HSC mimicry, stemness and EMT of MDA and 4T1 BrCa cells, Figure S6: Full agarose gel images for Figures S1 and S3, Figure S7: Full agarose gel images for Figures S4 and S5, Figure S8: Full agarose gel images for Figure S5, Table S1: Primer sequences, Table S2: Antibody information, Table S3: TaqMan™ Array Mouse Stem Cell Pluripotency gene expression, Table S4: Breast Cancer tissue array donor information.
