# Supplementary material for: Role of Neural (N)-Cadherin in Breast Cancer Cell Stemness and Dormancy in the Bone Microenvironment
Source: Cancers (Basel). 2022 Mar 4;14(5):1317. doi: 10.3390/cancers14051317 (PMC8909418; doi:10.3390/cancers14051317)
Supplement: Supplementary file 1 [file cancers-14-01317-s001.zip › cancers-1444963-supplementary.pdf]

Supplementary Materials

Role of Neural (N)-Cadherin in Breast Cancer Cell Stemness and Dormancy in the Bone Microenvironment

Antonio Maurizi, Michela Ciocca, Cristiano Giuliani, Ilaria Di Carlo and Anna Teti

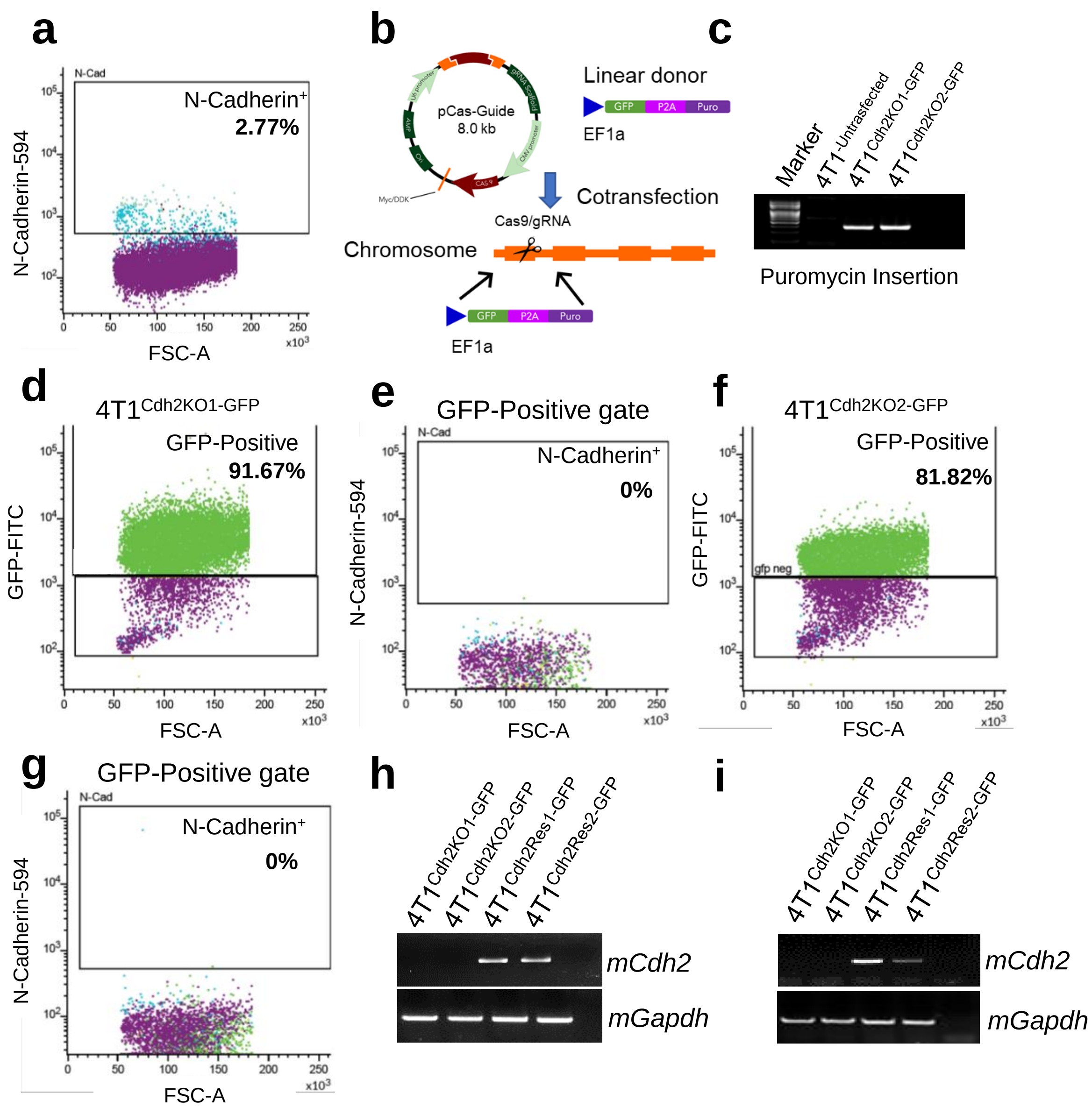

Figure S1. Generation of genetically modified 4T1 cells. (**a**) Intact 4T1 mouse BrCa cells were stained using a specific antibody for the N-Cadherin and the percentage of positive cells was assessed by flow cytometry. (**b**) Schematic representation of the CRISPR/Cas9 strategy used to generate the N-Cadherin knock-out (KO) 4T1 cells. (**c**) The integration of linear donor was evaluated in the 4T1$^{Cdh2KO1\text{-}GFP}$ and 4T1$^{Cdh2KO2\text{-}GFP}$ by RT-PCR using a specific primer pair for Puromycin. (**d–g**) 4T1$^{Cdh2KO1\text{-}GFP}$ and 4T1$^{Cdh2KO2\text{-}GFP}$ were stained with a specific antibody for N-Cadherin and the percentage of the positive cells was measured in the GFP-positive gate to validate the success of the CRISPR/Cas9 gene editing. (**h**) 4T1$^{Cdh2KO1\text{-}GFP}$ and $^{4T1Cdh2KO2\text{-}GFP}$ were stably transfected using *Cdh2*-turboGFP expression vector (4T1^{Cdh2Res1tGFP} and 4T1$^{Cdh2Res2\text{-}GFP}$) or with the empty-turboGFP vector (4T1$^{Cdh2KO1\text{-}GFP}$ and 4T1$^{Cdh2KO2\text{-}GFP}$). The restored N-Cadherin expression was analysed by RT-PCR using specific primers for the mouse *Cdh2*. Gene expression was normalized by mouse *Gapdh*. Pictures are representative of 3 experiment or 3 cell preparations. Full agarose gel images available in Figure S6.

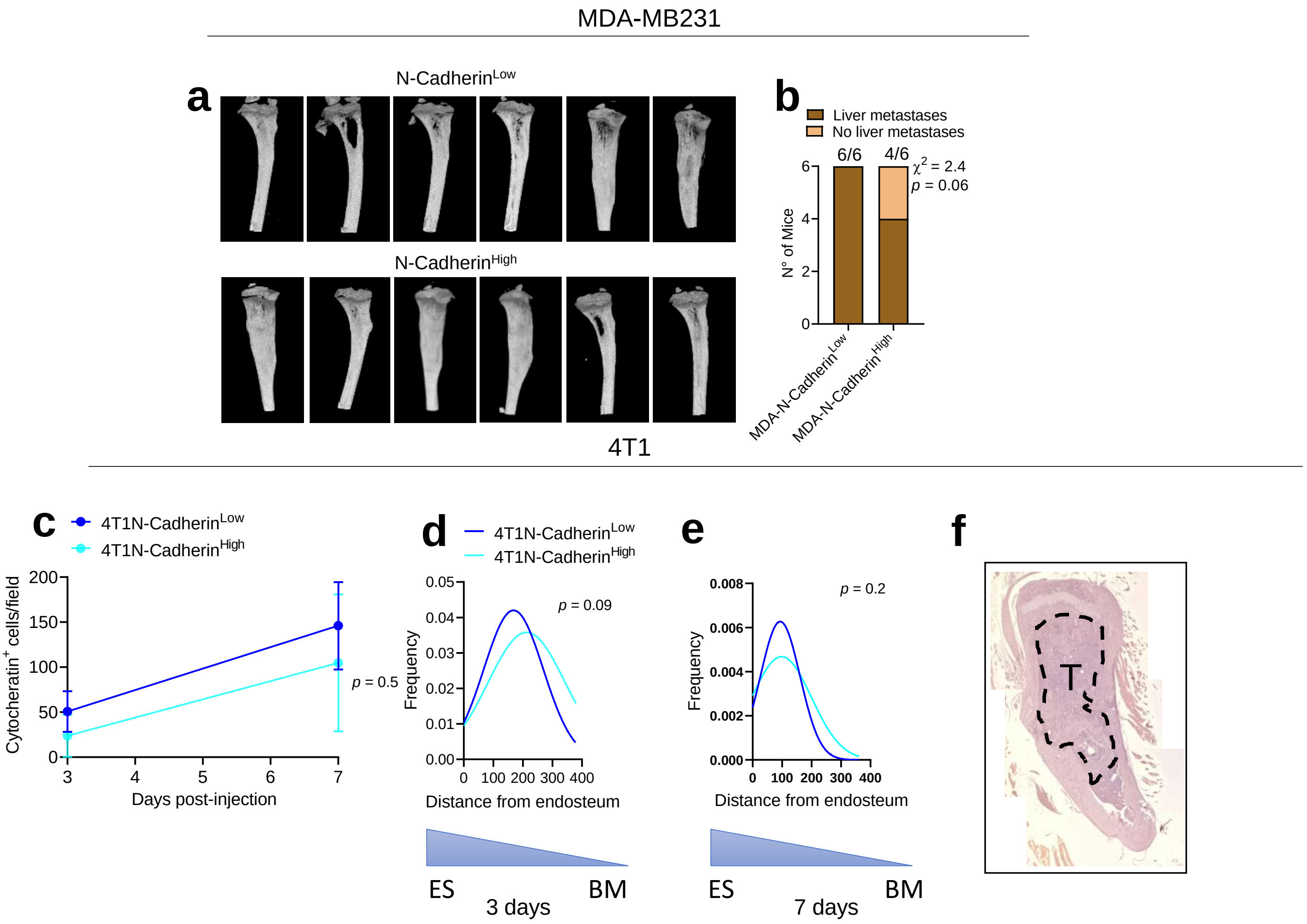

Figure S2. In vivo analysis of MDA- and 4T1-N-Cadherin^{High} BrCa cells. Four-week old Balb-c *nu*/*nu* female mice were intratibially injected with 1×10^4 MDA cells MACS-sorted in N-Cadherin^{High} and N-Cadherin^{Low} subpopulations. (**a**) 3D µCT representative pictures of all mice injected. (**b**) Liver metastases incidence. (**c**) Four-week old Balb-c wild type female mice were intratibially injected with 1×10^4 4T1 cells MACS-sorted into N-Cadherin^{High} and N-Cadherin^{Low} subpopulations and sacrificed after 3- and 7-days post-injection. Paraffin-embedded tibias harvested from the injected mice were immunostained for cytokeratin to visualise the tumour cells in the bone tissue. Total number of cytokeratin positive 4T1 cells and (**d**) cell distribution in relation to the endosteum were measured after 3 and (**e**) 7 days. (**f**) Representative H&E picture of paraffin-embedded tibia harvested from Balb/C wild type female mice injected with 1×10^4 4T1 cells and sacrificed after 10 days post-injection. Data are the mean ± SD and pictures are representative of 4–6 mice per group. Statistical analysis: (**b**) χ square analysis, (**c**) linear regression fitting and *F*-test, (**d**,**e**) Gaussian curve regression fitting and *F*-test.

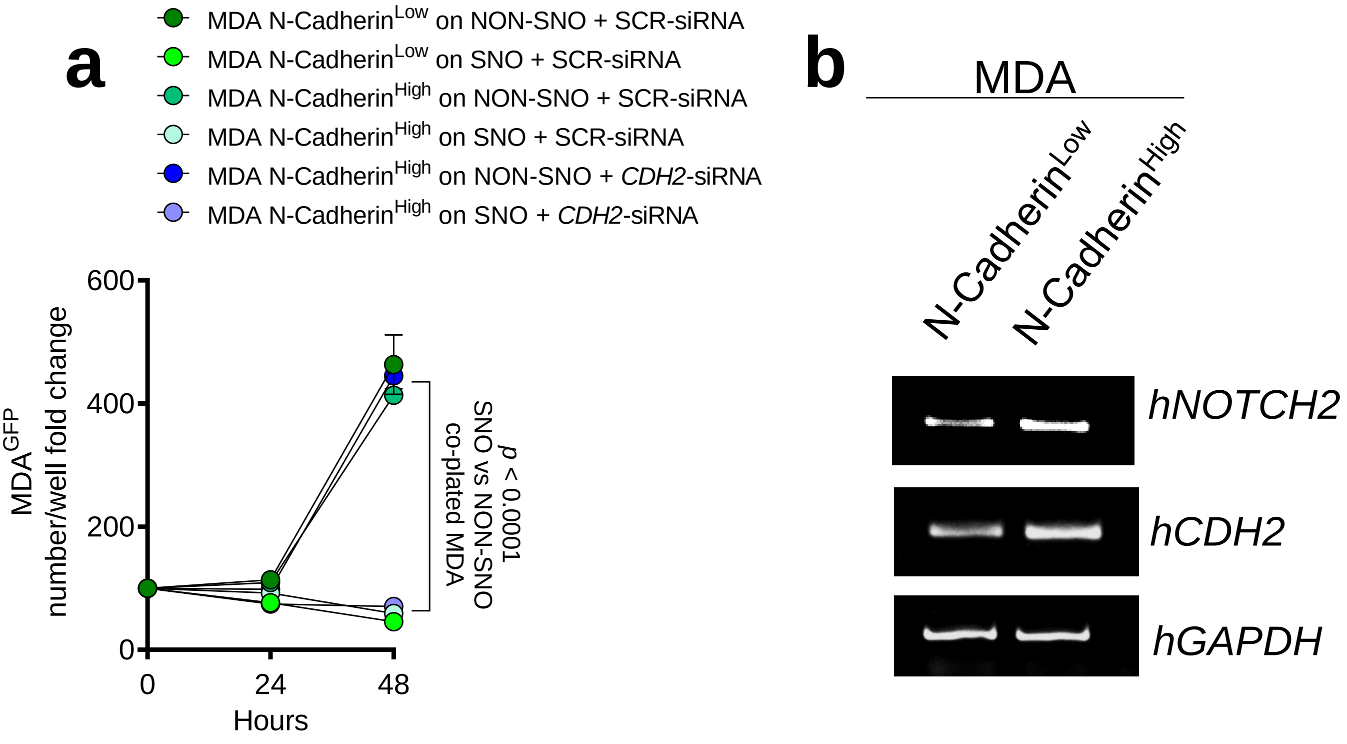

Figure S3. Effect of N-Cadherin down regulation on proliferation to SNOs and NON-SNOs and Notch2/N-Cadherin co-expression analysis in MDA cells. (**a**) N-Cadherin^{High} and N-Cadherin^{Low} MDA^{GFP} cells treated with siRNA against the N-Cadherin (*CDH2*-siRNA) or scrambled (SCR-siRNA) for 48 hours were seeded onto MACS-sorted SNOs and NON-SNOs and allowed to attach for 1 h at 37 °C, followed by extensive washing. The number of MDA^{GFP} cells was assessed after 24–48 h of co-culture. (**b**) Semiquantitative RT-PCR was used to assess the expression of the indicated genes. Human *GAPDH* was used to normalise the gene expression. Pictures are representative and data are the mean ± SD of 3 independent cell sorting. Statistical analysis: (**a**) Non-linear regression fitting and *F*-test. Full agarose gel images available in Figure S6.

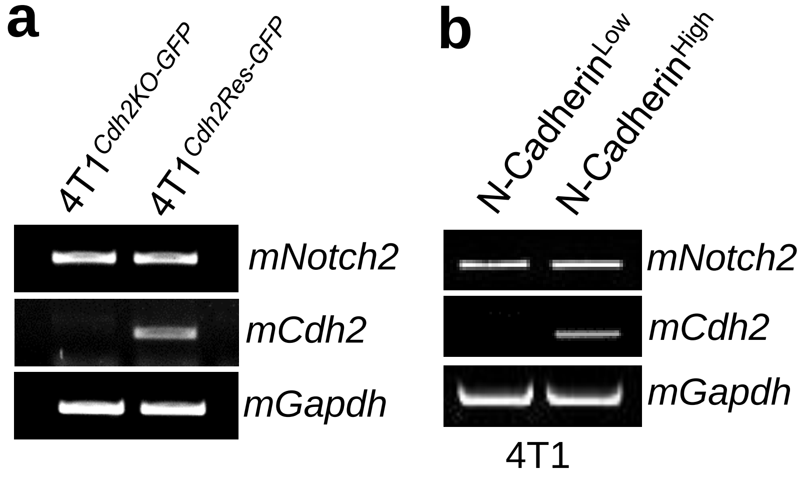

Figure S4. Notch2 and N-Cadherin co-expression analysis in 4T1 cells. Semiquantitative RT-PCR to assess the expression of the indicated genes in (**a**) 4T1$^{Cdh2KO-GFP}$ and 4T1$^{Cdh2Res-GFP}$ or (**b**) N-Cadherin^{High} and N-Cadherin^{Low} MACS-sorted 4T1 cells. Mouse *Gapdh* was used to normalise gene expression. Pictures are representative of 3 independent cell preparations or cell sorting. Full agarose gel images available in Figure S7.

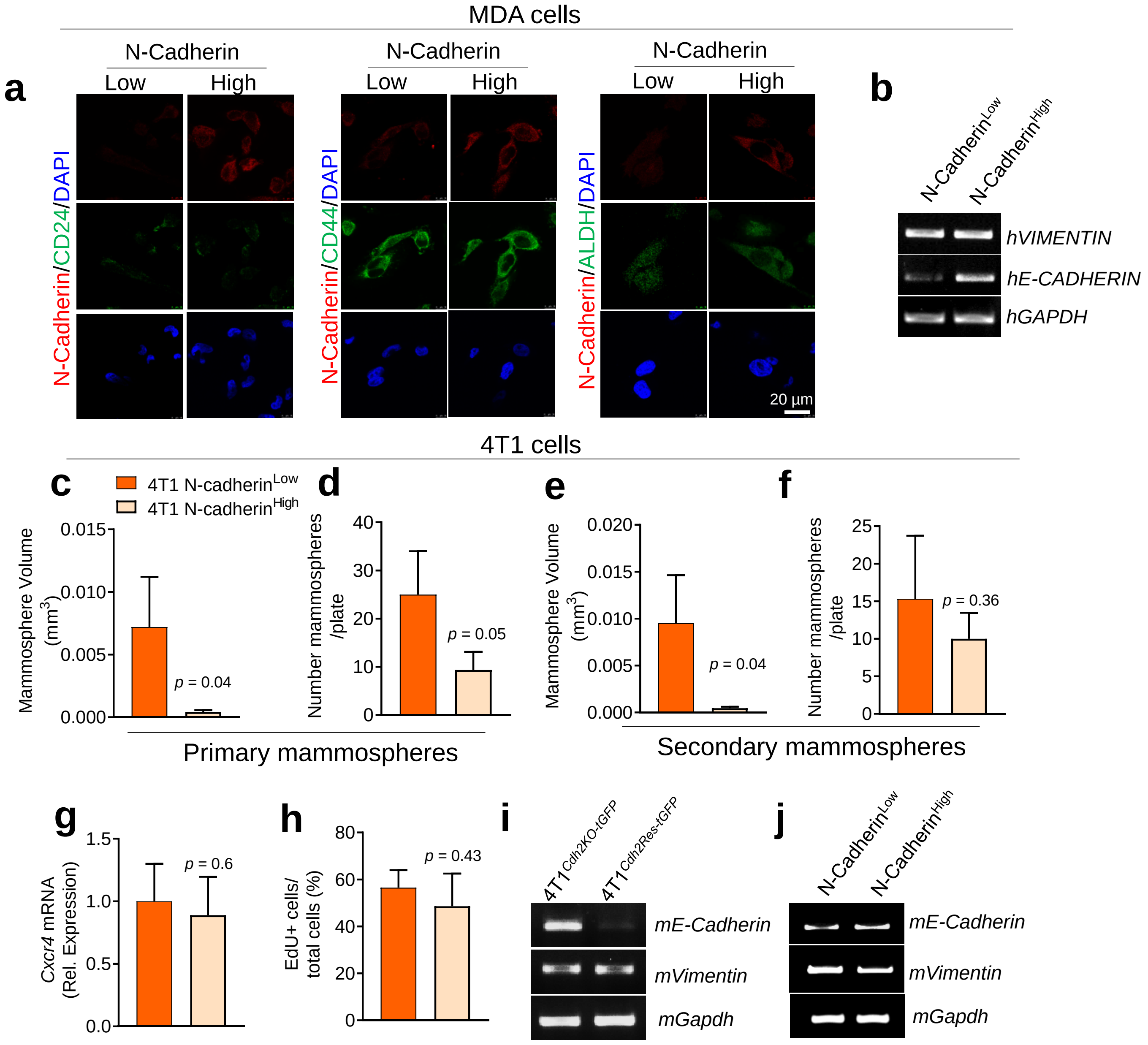

Figure S5. Effect of N-Cadherin expression on HSC mimicry, stemness and EMT of MDA and 4T1 BrCa cells. (**a**) MDA cells were sorted into N-Cadherin^{High} and N-Cadherin^{Low} subpopulations by MACS and immunofluorescence was performed using the indicated antibodies. (**b**) Semiquantitative RT-PCR to assess the expression of the indicated EMT genes. Human *GAPDH* was used to normalise gene expression. (**c**) Size and (**d**) number of primary mammospheres obtained from MACS-sorted N-Cadherin^{High} and N-Cadherin^{Low} 4T1 cells. (**e**) Size and (**f**) number of secondary mammospheres obtained after trypsinization and re-plating of single cells harvested from the primary 4T1 mammospheres. (**g**) Mouse *Cxcr4* gene ex-pression assessed by real time RT-PCR. (**h**) 5-ethynyl-2′-deoxyuridine (EdU) assay to assess cell proliferation. (**i**) Semi-quantitative RT-PCR to assess the expression of the indicated EMT genes in MACS-sorted N-Cadherin^{High} and (**j**) N-Cad-herin^{Low} 4T1 or 4T1$^{Cdh2KO\text{-}tGFP}$ and 4T1$^{Cdh2Res\text{-}tGFP}$ cells. Mouse *Gapdh* was used to normalise gene expression. Pictures are representative and data are the mean ± SD of 3 independent cell preparations. Statistical analysis: Student's *t*-test. Full agarose gel images available in Figures S7 and S8.

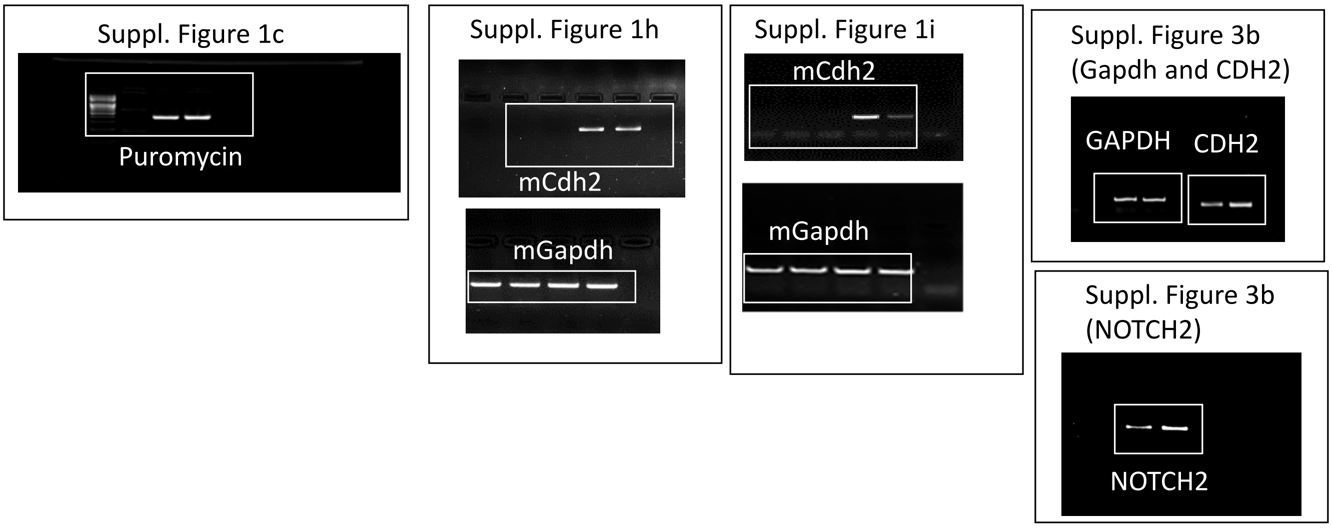

Samples are loaded as reported in the figures

Figure S6. Full agarose gel images for Figures S1 and S3.

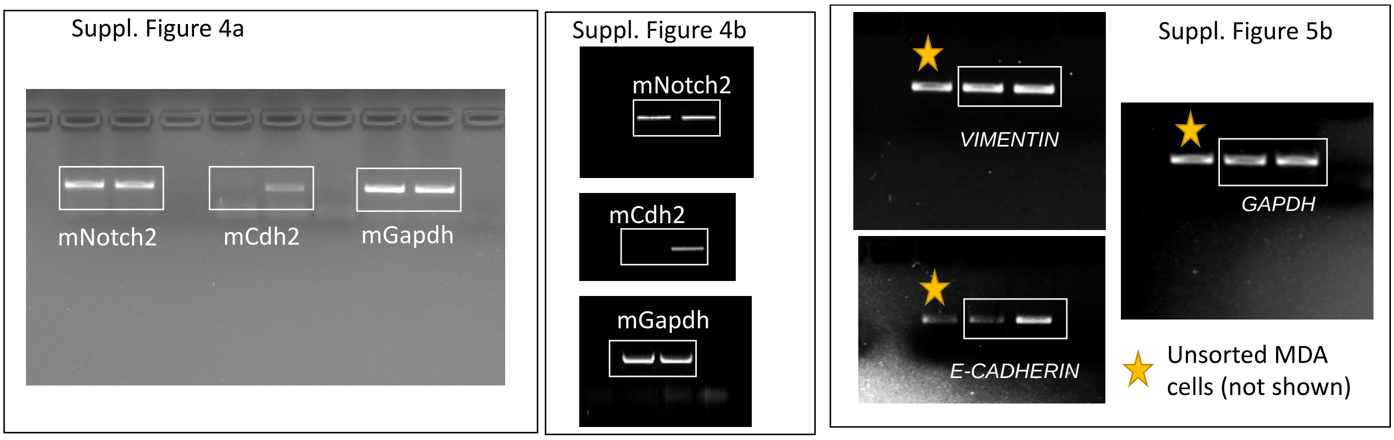

Samples are loaded as reported in the figures

Figure S7. Full agarose gel images for Figures S4 and S5.

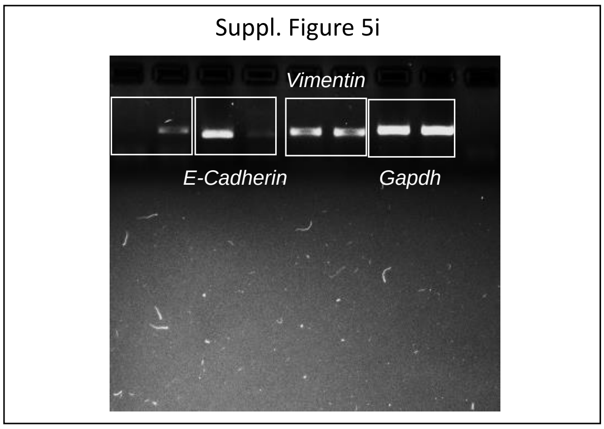

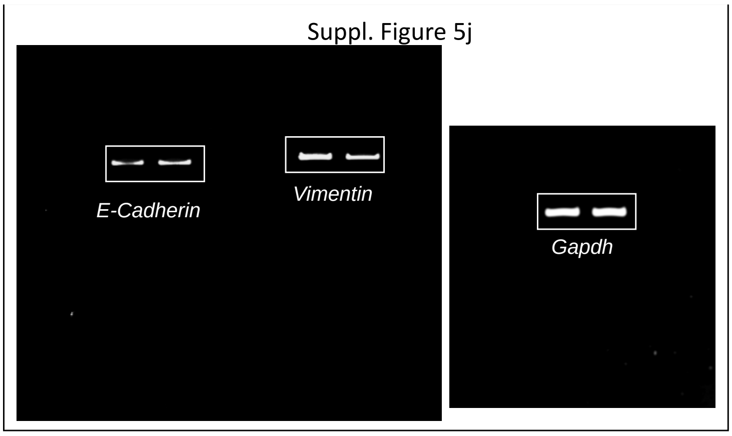

Samples are loaded as reported in the figures

Figure S8. Full agarose gel images for Figure S5.

Table S1. Primer sequences.

Primer Name	Sequences
**Human**	
GAPDH	Fw: CAATCTTCCAGGAGCGAGAT
	Rv: CAGTGATGGCATGGACTGTG
CDH2	Fw: CCATTAAGCCGAGTGATGGT
	Rv: GACAATGCCCCTCAAGTGTT
NOTCH2	Fw: CTGGAGTACAGGAGGCGAAG
	Rv: ATGACTGCCCTAACCACAGG
CXCR4	Fw: GACGCCAACATAGACCACCT
	Rv: CTGAGAAGCATGACGGACAA
CD34	Fw: GCCGAGTCACAATTCGGTAT
	Rv: GCAAGCCACCAGAGCTATTC
TIE-2	Fw: TGTGAAGCGTCTCACAGGTC
	Rv: CCAAACGTGATTGACACTGG
CYCLIND1	Fw: CCTTCCGGTGTGAAACATCT
	Rv: AGCGCTGTTTTTGTTGTGTG
VIMENTIN	Fw: GGCTCAGATTCAGGAACAGC
	Rv: GCTTCAACGGCAAAGTTCTC
E-CADHERIN	Fw: TGCCCAGAAAATGAAAAAGG
	Rv: GGATGACACAGCGTGAGAGA
CD24	Fw: AACTAATGCCACCACCAAGG
	Rv: CCTGTTTTTCCTTGCCACAT
CD44	Fw: GTGATCAACAGTGGCAATGG
	Rv: TCCAACGGTTGTTTCTTTCC
ALDH1A1	Fw: TGTTAGCTGATGCCGACTTG
	Rv: TTCTTAGCCCGCTCAACACT
ALDH1A2	Fw: TGATCCTGCAAACACTGCTC
	Rv: CTGGAGCTGGGTGGTAAGAG
ALDH1A3	Fw: TCTCGACAAAGCCCTGAAGT
	Rv: TATTCGGCCAAAGCGTATTC
SOX2	Fw: CGATGCCGACAAGAAAACTT

	Rv: TTTTTCGTCGCTTGGAGACT
C-MYC	Fw: TACCCTCTCAACGACAGCAG
	Rv: TCTTGAATTCTCCTCGGTG
NANOG	Fw: ACCCAGAACATCCAGTCCTG
	Rv: AGGAGGGGAGAGGAAGGATT
KLF4	Fw: TCTCCCACATGAAGCGACTT
	Rv: CGGATCGGATAGGTGAAGC
**Mouse**	
Gapdh	Fw: TGTGAGGGAGATGCTCAGTG
	Rv: TGTTCCTACCCCCAATGTGT
Cdh2	Fw: ATGACGTCCACCCTGTTCTC
	Rv: CTGGGACGTATGTGATGACG
Notch2	Fw: CCAGGTTATTGCACGTTCCT
	Rv: ACCCTTGTATGCACGGAGTC
Cxcr4	Fw: TTGCCGACTATGCCAGTCAAG
	Rv: TCCAACAAGGAACCCTGCTTC
CyclinD1	Fw: TCAAGTGTGACCCGGACTG
	Rv: ATGTCCACATCTCGCACGTC
Vimentin	Fw: TCGATGTAGTTGGCAAAGCG
	Rv: TCTGTGTCCTCGTCCTCCTA
E-Cadherin	Fw: CACATGCTCAGCGTCTCCTC
	Rv: GGTCTCTGGTCCCTTCCACA

Fw: forward, Rv: reverse.

Table S2. Antibody information.

Antibody	Dilution	Species	Cat #	Company
N-Cadherin	(IF)(IHC)1:100 (FACS)1:20	Mouse	NBP1-48309	Novus (Centennial, CO, USA)
N-Cadherin-PE	(FACS)1:50	Mouse	NBP1-48309PE	Novus (Centennial, CO, USA)
N-Cadherin	(FACS) 1:20	Rabbit	PA5-17526	ThermoFisher (Waltham, MA, USA)
Pan-Cytokeratin (AE1/AE3)	(IHC)(IF)1:100	Mouse	sc-81714	Santa Cruz Biotechnology
Ki67	(IF)1:500	Rabbit	MA5-14520	ThermoFisher (Waltham, MA, USA)
Notch2	(FACS)1:50	Rabbit	Sc-5545	Santa Cruz Biotechnology (Dallas, TX, USA)
CD24	(IF) 1:100	Mouse	555426	BD (Franklin Lakes, NJ, USA)
CD44	(IF) 1:100	Rabbit	sc-7946	Santa Cruz Biotechnology (Dallas, TX, USA)
ALDH	(IF) 1:100	Mouse	611194	BD (Franklin Lakes, NJ, USA)
AlexaFluor488 anti-mouse	(IF)1:500 (FACS) 1:50	Goat	A11001	Invitrogen (Waltham, MA, USA)
AlexaFluor488 anti-rabbit	(IF)1:500 (FACS) 1:50	Goat	A11008	Invitrogen (Waltham, MA, USA)
AlexaFluor594 anti-mouse	(FACS)1:50	Goat	A11005	Invitrogen (Waltham, MA, USA)
AlexaFluor594 anti-rabbit	(IF)1:500 (FACS) 1:50	Goat	A11037	Invitrogen (Waltham, MA, USA)

IF: Immunofluorescence; IHC: Immunohistochemistry; FACS: fluorescence-activated single cell sorting.

Table S3. TaqMan™ Array Mouse Stem Cell Pluripotency gene expression.

Gene Symbol	Expression in $4T1^{Cdh2Res\text{-}tGFP}$ (Fold vs. $4T1^{Cdh2KO\text{-}tGFP}$)	*p*-Value
Actc1	n.d.	-
Afp	n.d.	-
Bxdc2	0.50 ± 0.09	**0.007**
Cd34	n.d.	-
Cd9	0.79 ± 0.28	0.28
Cdh5	n.d.	-
Cdx2	n.d.	-
Col1a1	n.d.	-
Col2a1	n.d.	-
Commd3	0.69 ± 0.31	0.16
Crabp2	n.d.	-
Ddx4	n.d.	-
Des	n.d.	-
Dnmt3b	0.54 ± 0.16	**0.008**
Lefty1	n.d.	-
Eomes	7.16 ± 10.18	0.35
Fgf4	n.d.	-
Fgf5	n.d.	-
Flt1	0.77 ± 0.18	0.10
Fn1	0.74 ± 0.28	0.20
Foxa2	n.d.	-
Foxd3	n.d.	-
Gabrb3	n.d.	-
Gal	n.d.	-
Gata4	0.57 ± 0.30	**0.007**
Gata6	n.d.	-
Gbx2	n.d.	-
Gcg	n.d.	-
Gcm1	n.d.	-
Gdf3	n.d.	-
Gfap	n.d.	-
Grb7	0.21 ± 0.13	**0.0005**
Hbb-b2	n.d.	-
Hba-x	n.d.	-
Mnx1	n.d.	-
Iapp	n.d.	-
Ifitm1	1.29 ± 0.45	0.32
Ifitm2	0.65 ± 0.26	0.08
Il6st	0.55 ± 0.19	**0.01**
Igfbp2	n.d.	-
Ins2	n.d.	-
Pdx1	n.d.	-
Isl1	n.d.	-
Kit	n.d.	-
Krt1	n.d.	-
Lama1	n.d.	-
Lamb1-1	0.54 ± 0.24	**0.03**
Lamc1	0.57 ± 0.23	**0.03**
Lefty2	n.d.	-
Lifr	0.83 ± 0.23	0.30
Lin28	n.d.	-
Myf5	n.d.	-
Myod1	n.d.	-
Nanog	n.d.	-
Nes	0.72 ± 0.49	0.40

Neurod1	n.d.	-
Nodal	n.d.	-
Nog	0.28 ± 0.25	**0.008**
Nppa	n.d.	-
Nr5a2	n.d.	-
Nr6a1	0.61 ± 0.28	0.07
Olig2	n.d.	-
Pax4	n.d.	-
Pax6	n.d.	-
Pecam1	n.d.	-
Podxl	0.86 ± 0.35	0.68
Pou5f1	n.d.	-
Pten	0.84 ± 0.35	0.50
Ptf1a	n.d.	-
Rest	0.64 ± 0.26	0.08
Runx2	n.d.	-
Sema3a	1.90 ± 1.83	0.44
Serpina1a	n.d.	-
Sfrp2	n.d.	-
Sox17	n.d.	-
Sox2	n.d.	-
Sycp3	n.d.	-
Syp	n.d.	-
T	n.d.	-
Tat	n.d.	-
Tdgf1	n.d.	-
Tert	0.56 ± 0.19	**0.01**
Tcfcp2l1	n.d.	-
Th	n.d.	-
Utf1	n.d.	-
Wt1	n.d.	-
Xist	n.d.	-
Zfp42	n.d.	-
Eras	n.d.	-
Raf1	1.17 ± 0.70	0.70
Ctnnb1	0.67 ± 0.30	0.13
Eef1a1	0.1.05 ± 0.87	0.92

n.d.: not detectable. Significant *p*-values are highlighted in bold.

Table S4. Breast cancer tissue array donor information.

Patient #	N-Cadherin$^+$ cells (n/mm^2)	Grade of Differentiation	TNM [1]	ER	PR	Her2
A3	0.268754733	moderately	T2N2M1	−	−	++
A4	0.489416875	moderately	T2N2M1	−	−	−
A5	0.578140816	moderately	T2N1M0	+	−	−
A6	1.519303172	moderately	T2N2M1	+	+	−
A7	1.172902049	moderately	T2N1M1	−	−	−
A8	0.717111097	moderately	T2N0M0	−	−	−
A9	1.045280582	moderately	T2N0M0	+	++	−
A10	1.238009699	moderately	T2N0M0	++	+	−
A11	0.613798482	moderately	T2N2M1	+	−	−
B1	1.002740094	moderately	T2N0M0	+++	+++	−
B2	1.780623318	moderately	T2N0M0	−	−	−
B3	0.996662881	well	T2N2M0	−	−	−
B4	0.990585668	well	T2N2M0	+	+	−
B5	0.237011295	moderately	T2N1M0	+++	++	−
B6	0.753574373	moderately	T2N2M0	−	+++	−
B7	0.929813541	moderately	T2N1M1	−	−	−
B8	1.294446303	moderately	T2N1M1	−	−	−
B9	0.583412418	poorly	T3N3M0	++	+	−
B10	0.559103567	moderately	T2N1M0	−	−	−
B11	1.02450169	moderately	T2N2MO	−	−	−
C1	0.301716335	moderately	T2N1M0	++	+	−
C2	0.303860634	moderately	T2N0M0	−	−	−
C3	0.47161648	moderately	T2N1M1	+++	−	−
C4	0	moderately	T2N3M1	−	−	++
C5	0.820423713	moderately	T2N2M0	−	−	−
C6	0.924575038	poorly	T2N0M0	++	+	−
C7	1.140809202	poorly	T2N0M0	+	+	−
C8	0.765728799	N/A	T2N1M1	−	−	−
C9	0.653817617	N/A	T1N0M0	−	−	−
C10	1.482839896	N/A	T2N3M1	+	−	−
C11	1.237246384	N/A	T1N0M0	+	+	++
D1	0.88119584	N/A	T2N0M0	+	+	−
D2	0.929813541	moderately	T2N2M0	+	+	−
D3	1.317934063	poorly	T2N0M0	+	++	−
D4	0.963586355	poorly	T2N1M0	++	+++	−
D5	1.173030367	moderately/poorly	T2N1M0	−	−	−
D6	0.814435591	moderately	T2N3M1	−	−	−
D7	0.626021387	moderately	T3N3M0	+	+	−
D8	1.023707197	moderately	T2N0M0	−	−	−
D9	1.13253033	poorly	T2N0M0	−	−	−
D10	0.807312152	poorly	T2N0M0	−	−	−
D11	0.784046204	poorly	T2N0M0	−	−	−
E1	1.291734397	moderately	T2N0M0	−	−	−
E2	0.899525877	moderately	T2N0M0	−	−	−
E3	0.632099265	moderately	T2N0M0	−	+	−
E4	0.583476244	moderately	T2N0MO	−	+++	−
E5	0.634051873	moderately	T2N0MO	−	−	−
E6	0.32212751	moderately	T2N2M1	+	+	−
E7	0.522697469	moderately	T2N0M0	−	−	+++
E8	1.258120652	moderately	T2N0M0	−	−	+++
E9	0	moderately	T2N0M0	−	−	+++
E10	0.819838504	moderately	T2N0M0	−	−	+++
E11	0.613865632	moderately	T2N0M0	−	+	+++
F1	0.480152326	moderately	T2N3M0	−	−	−
F2	1.166952489	poorly	T2N1M0	−	−	+
F3	0.455840816	moderately	T2N0M0	−	−	+++

F4	0.632099265	moderately	T2N0M0	−	−	+++
F5	0.492308081	moderately	T2N0M0	−	−	−
F6	0.200569959	moderately	T2N1M0	+	+	−
F7	0	moderately	T2N0M0	−	−	−
F8	0.747578938	moderately	T2N0M0	−	−	−
F9	0.121557551	moderately	T2N0M0	−	−	−
F10	0.467996571	moderately	TxNxMx	−	−	−
F11	0.583476244	moderately/poorly	TxNxMx	+	+	−

[1] TNM staging: T1: tumour is 2 centimetres (cm) across or less. T2: tumour is more than 2 centimetres but no more than 5 centimeters across. T3: tumour is bigger than 5 centimetres across. TX: the tumour size can't be assessed. N0: No cancer was found in the lymph nodes or only areas of cancer smaller than 0.2 mm are in the lymph nodes. N1: cancer has spread to 1 to 3 axillary lymph nodes and/or the internal mammary lymph nodes. N2: cancer has spread to 4 to 9 axillary lymph nodes. Or, it has spread to the internal mammary lymph nodes, but not the axillary lymph nodes. N3: cancer has spread to 10 or more axillary lymph nodes, or it has spread to the lymph nodes located under the clavicle, or collarbone. M0: there is no sign that the cancer has spread (No distal metastases). M1: cancer has spread to another part of the body (Distal metastases). "−":negative; "+": weak positivity; "++": intermediate positivity,"+++": strong positivity.

© 2022 by the authors. Licensee MDPI, Basel, Switzerland. This article is an open access article distributed under the terms and conditions of the Creative Commons Attribution (CC BY) license (http://creativecommons.org/licenses/by/4.0/).
